# Assessment of Reporting Quality in Randomized Controlled Trials of Acupuncture for Primary Insomnia with CONSORT Statement and STRICTA Guidelines

**DOI:** 10.1155/2022/5157870

**Published:** 2022-02-17

**Authors:** Jinsong Yang, Fanjun Yu, Keyi Lin, Haotian Qu, Yihan He, Jing Zhao, Fen Feng, Litao Pan, Yu Kui

**Affiliations:** ^1^Guangzhou University of Chinese Medicine, Guangzhou, China; ^2^Guangdong Provincial Academy of Chinese Medical, Guangzhou, China; ^3^The Second Clinical Medical College of Guangzhou University of Chinese Medicine, Guangzhou, China; ^4^Institute of Chinese Medical Science, University of Macau, Macau, China; ^5^Hospital of Chengdu University of Traditional Chinese Medicine, Chengdu, China; ^6^The First Affiliated Hospital of Shenzhen University, Shenzhen, China

## Abstract

**Aim:**

To assess the reporting quality of randomized controlled trials (RCTs) on acupuncture for primary insomnia (PI).

**Methods:**

Seven Chinese and English databases were searched for publication reporting RCTs on acupuncture for PI from the inception of the databases to August 6, 2021. The internationally recognized Consolidated Standards of Reporting Trials (CONSORT) statement and the International Standards for Reporting Interventions in Controlled Trials of Acupuncture (STRICTA) guidelines were used to evaluate the reporting quality. The agreement between two researchers was calculated by Cohen's kappa.

**Results:**

A total of 102 eligible RCTs were assessed. According to the CONSORT statement (2017), the positive reporting rates of items such as “abstract,” “background,” “participants,” and “numbers analyzed” were above 80%. However, the positive reporting rates of items such as “sample size,” “randomization implementation,” “Outcomes and estimation,” “Ancillary analyses,” and “Registration” were below 20%. According to STRICTA guidelines, the positive reporting rates of items such as “style of acupuncture,” “reasons for acupuncture treatment,” “Number of needles inserted,” “Needle retention time,” “Treatment regimen,” and “precise description of the control intervention” were above 80%. However, the positive reporting rates of items such as “setting and context of treatment” and “practitioner background” were below 20%.

**Conclusion:**

It is essential to advocate the endorsement of the CONSORT statement and STRICTA guidelines to improve the quality of acupuncture RCT reports.

## 1. Introduction

Primary insomnia (PI) is characterized by difficulty in falling asleep or staying asleep, with the exclusion of insomnia caused by various secondary factors including mental, physical, and neurological disorders, alcohol, or drugs [1]. The overall prevalence of insomnia was 5%–10% worldwide [2], while the incidence rate of insomnia in females is higher than that in males [3]. Severe insomnia affects normal life and work and increases the risk of various diseases [4].

At present, the treatments of insomnia mainly include cognitive behavioral therapy (CBT), drug therapy, physical therapy, and complementary and alternative medicine (CAM) [5]. CBT, the first-line therapy of insomnia, costs a lot and lacks effective training providers, making it difficult to popularize [6]. Although the short-term efficacy of drugs in the treatment of insomnia has been confirmed, it has been reported that there are side effects such as hangover, drug resistance, and drug dependence [5]. The efficacy of physical therapy (such as transcranial magnetic stimulation and phototherapy) is not certain yet [4, 7]. Acupuncture in CAM has been applied by acupuncture practitioners and accepted by patients and is being increasingly widely used to treat insomnia [8].

The number of RCTs of acupuncture for PI has been increasing gradually in recent years, but their work varies considerably in quality [9]. Accurate and standardized RCT reports not only can reduce the bias of systematic evaluation but also help medical decision-making [10]. With high-quality reports, acupuncture practitioners can easily master effective operating procedures [11].

In the past ten years, there has not been any study on the quality evaluation of RCTs of acupuncture for PI. The CONSORT statement (https://www.consort-statement.org/) is an evidence-based report guide designed to improve research transparency and reduce waste [10]. STRICTA (https://stricta.info/), an independent guide for reporting acupuncture research, has become a formal extended version of the CONSORT report of RCTs [11]. These guidelines ensure transparency and a coherent approach to testing and reporting trials of complex interventions [10]. A range of studies have shown that it can help readers gain information on study design, intervention implementation, data analysis, and so on [12, 13]. Therefore, in this study, the internationally recognized CONSORT statement of nondrug RCTs (2017) and STRICTA were used to evaluate the reporting quality of RCTs of acupuncture for PI so as to lay a foundation for high-quality RCT design of acupuncture treatment for insomnia in the future.

## 2. Methods

### 2.1. Document Retrieval Methods

(1) Database for retrieval: China National Knowledge Infrastructure (CNKI), China Science and Technology Journal Database (VIP), Wanfang Database (WF), PubMed, Embase, Web of Science, and Cochrane Library. (2) Publication time: from database creation to August 6, 2021. (3) Search strategy: the following search terms were used in both Chinese and English: (primary insomnia) AND (acupuncture OR acupuncture therapy) AND (randomized controlled trial OR RCT).

### 2.2. Inclusion Criteria

All the following criteria were met: (1) confirmed diagnosis of PI; (2) RCT of acupuncture to treat PI; and (3) the intervention measures of the experimental group were acupuncture (including hand acupuncture, electroacupuncture, ear acupuncture, abdominal acupuncture, and eye acupuncture) or acupuncture combined with traditional Chinese medicine (TCM) nondrug therapy other than acupuncture (acupressure, moxibustion, ear point treatment, etc.); the control group was treated with Western medicine or sham acupuncture or acupuncture at nonmeridian and nonacupoints.

### 2.3. Exclusion Criteria

One of the following criteria was met: (1) not in accordance with the inclusion criteria; (2) no confirmed diagnosis of PI in the RCT (such as diagnosis of perimenopausal insomnia); (3) in addition to acupuncture, the intervention measures in the experimental group also included drug treatment; (4) the control group was treated with routine acupuncture; (5) if the content of conference paper and periodical paper or Chinese paper and English paper were similar, the one with higher quality were chosen; (6) if the papers were about the same RCT, the one published later was chosen; (7) the paper was about research protocol; and (8) full-text paper not available.

### 2.4. Document Screening and Data Extraction

Paper screening, data extraction, and cross-checking were performed by two researchers independently. In case of any disagreement, the two researchers negotiated first. Firstly, the RCT was imported into NoteExpress 3.4 (Guangzhou University of Chinese Medicine library version, released 2021, Beijing, China). After duplicate checking, the title and abstract of the paper were read. After excluding the clearly irrelevant papers, the full text of the paper was read to determine whether the inclusion should be made. The extracted data included the year of publication, author, intervention measures, CONSORT statement (2017), STRICTA standard entries, and relevant information, which were entered into Excel 2016. Disagreements between the two researchers over data selection and extraction were resolved by discussion, with the involvement of a third researcher (Yu Kui).

### 2.5. Quality Evaluation Method

25 items from CONSORT (2017) and 6 items from STRICTA were used to evaluate the quality of reports. Two trained researchers with no interest conflicts extracted the data independently and evaluated the report item by item as “reported,” “partially reported,” or “not reported.” Then, their results were cross-checked. In case of disagreement, a third researcher with no interest conflicts should arbitrate. The number of RCTs meeting each item was calculated, and the percentage of items reported was also calculated.

Cohen's *к*-statistic was calculated to assess the agreement between two researchers. We judged agreement as poor if *к* ≤ 0.2; fair if 0.2 < *к* ≤ 0.4; moderate if 0.4 < *к* ≤ 0.6; substantial if 0.6 < *к* ≤ 0.8; good if 0.8 < *к* < 1; and prefect if *к* = 1 [14]. Cohen's *к*-statistic and 95% CI of each item were performed by using PASW Statistics for Windows, version 18.0 (SPSS Inc., Released 2009, Chicago, USA).

## 3. Results

### 3.1. Document Retrieval Results

**Figure 1:**
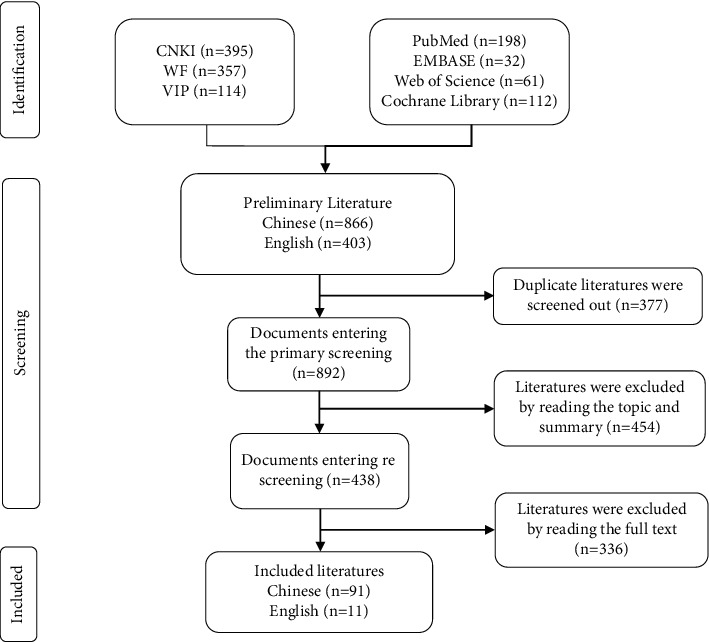
Process and results of literature screening.

According to the above retrieval strategies, a total of 1269 relevant RCTs were detected. Among them, 377 duplicated ones were screened out, and 454 clearly irrelevant ones were excluded by titles and abstracts. After reading the full text, 102 in accordance with the inclusion criteria were included, and 336 were excluded according to the exclusion criteria. Finally, 91 Chinese papers and 11 English papers were included. The process and results of literature screening are shown in Figure 1.

### 3.2. Information of Published Papers per Year

**Figure 2:**
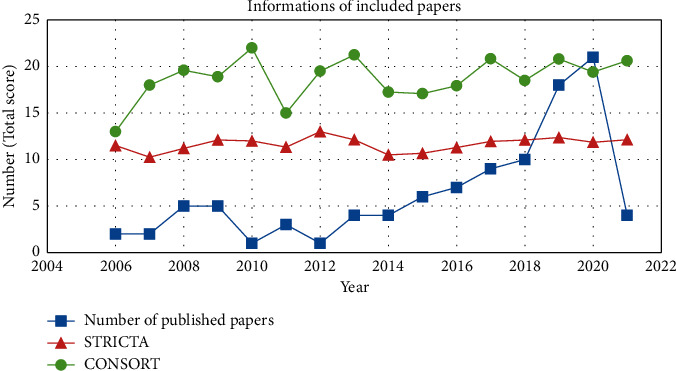
Information of included papers (*n* = 102).

For the 102 RCTs, the publication time was from 2006 to 2021. The number of RCTs published before 2014 was no more than five, and it increased year by year since 2015, indicating that acupuncture for PI is becoming the focused topic. Overall, the total score of CONSORT and STRICTA is on the rise. The average CONSORT and STRICTA total scores and the number of papers published in each year are shown in Figure 2.

### 3.3. CONSORT Statement (2017)

The quality of the included RCTs was assessed by the CONSORT statement (2017), as shown in Table 1. The positive reporting rates of items such as “abstract,” “background,” “participants,” and “numbers analyzed” were above 80%. However, the positive reporting rates of items such as “sample size,” “randomization implementation,” “Outcomes and estimation,” “Ancillary analyses,” and “Registration” were below 20%.

#### 3.3.1. Title, Abstract, and Introduction

Of the 102 papers included, 15 (14.7%) can be identified as RCTs by title (*к* = 1); 102 (100%) were structured abstracts including experimental design, methods, results, and conclusions (*к* = 1); 98 (96.1%) described the scientific background and made a reasonable explanation (0.57 < *к* < 1.13); and 102 (100%) were referred to specific purposes or assumptions (*к* = 1).

#### 3.3.2. Trial Methods

Among the 102 papers included, all (100%) described the experimental design and distribution proportion, and none made any changes in experimental methods after the beginning of the experiment (*к* = 1). All (100%) described the eligibility criteria of the participants (*к* = 1), and only 2 (2%) did not mention the place for data collection (0.04 < *к* < 1.28). A vast majority (99%) described the details of interventions for each group (*к* = 1). Among them, 12 (11.8%) described whether and how the interventions were standardized (0.59 < *к* < 0.95), while 5 (4.9%) mentioned whether the measure providers' compliance to the protocol was evaluated or how to enhance their compliance (0.42 < *к* < 0.98), and none (0%) mentioned whether the participants' compliance to the interventions was evaluated or how to enhance that (*к* = 1). 87 (85.3%) completely and accurately explained the primary and secondary outcome indicators (0.69 < *к* < 0.99), and the remaining 15 (14.7%) used only one outcome indicator. One article (1%) changed the outcome indicators and explained the reasons after the start of the trial (*к* = 1). 14 (13.7%) reported how to determine the sample size (0.73 < *к* < 1.01), and 1 (1%) partially reported. 17 articles (16.7%) described the principles of interpretation, analysis, and test suspension if there were corresponding situations (0.57 < *к* < 0.89).

#### 3.3.3. Randomized Methods

69 (67.6%) described the methods of generating random allocation sequences (0.77 < *к* < 0.97), and 4 (3.9%) mentioned the types of random methods and limited details (0.22 < *к* < 1.10). 34 (33.3%) mentioned the mechanism for performing random sequence allocation and described the steps of concealing sequence numbers (0.82 < *к* < 1.00). 10 (9.8%) reported who generated the random allocation sequence, included the participants, and assigned concealment to the participants (0.86 < *к* < 1.04), while 1 (1%) partially mentioned the abovementioned information. 23 (22.5%) mentioned the implementation of the blind method, to whom it was implemented and how the implementation was carried out (0.79 < *к* < 0.99). In 9 (8.8%), intervention measures were found to have similarities in case of relevant situations (0.61 < *к* < 0.99). 7 (6.9%) described the measures to limit bias in case the blind method could not be implemented (0.58 < *к* < 1.06). 100 (98%) reported the statistical methods used to compare the primary and secondary outcome indicators of each group (0.43 < *к* < 0.95), and 2 (2%) described the methods of additional analysis (*к* = 1).

#### 3.3.4. Trial Results

All the 102 RCTs included described the number of cases randomly assigned to each group, received the assigned treatment, and included in the analysis of outcome indicators (*к* = 1). Among them, 33 (32.4%) reported the number of cases dropped out and eliminated from each group and explained the reasons (0.84 < *к* < 0.98); 10 (9.8%) reported the number of cases dropped out and eliminated from each group but without explanation of the reasons; only 4 (3.9%) reported the time from random allocation to the delay of intervention implementation in each group (0.57 < *к* < 1.13).

#### 3.3.5. Intervention Implementation

Among the 102 papers, 55 (53.9%) reported the specific periods of recruitment and follow-up (0.75 < *к* < 0.95); 41 (40.2%) only reported the recruitment period but not the follow-up period; 6 (5.9%) mentioned neither. None reported suspension of the trial (*к* = 1). 62 (60.8%) listed the baseline data, demographic data, and clinical characteristics of participants of each group in one table (*к* = 1), and 37 (36.3%) only described in words. All papers mentioned the number of participants included in each analysis and whether they were analyzed according to the initial grouping (*к* = 1); only 3 (2.9%) explicitly mentioned the use of intention to treat (ITT) analysis. Only 5 (4.9%) mentioned the results of primary and secondary outcome indicators (0.65 < *к* < 1.11) and estimated effect size and relative effect value. 2 (2%) used correction analysis (*к* = 1). 45 (44.1%) reported all serious harmful or unintended effects in each group (0.91 < *к* < 1.01).

#### 3.3.6. Limitations, Generalizability, and Interpretation of Trial

Among the 102 papers included, 47 (46.1%) described the potential sources of the report bias, imprecision,s and multiple analysis (0.82 < *к* < 0.98); 65 (63.7%) described the generalizability of the trial findings (0.79 < *к* < 0.97); 50 (49%), provided the interpretation corresponding to the results, weighed the advantages and disadvantages of the results (0.80 < *к* < 0.98), and considered other relevant evidence, while another 50 (49%) partially included these.

#### 3.3.7. Other Information

Of the 102 papers included, only 12 (11.8%) included the clinical trial registration number and the name of the trial registry (*к* = 1). 36 (35.3%) provided the author's mailbox with possible access to the complete trial protocol (*к* = 1). 47 (46.1%) provided information of the sources of funding and other support (*к* = 1).

### 3.4. STRICTA Guidelines

STRICTA guidelines were used to assess the quality of included RCT reports, as shown in Table 2. The positive reporting rates of items such as “style of acupuncture,” “reasons for acupuncture treatment,” “number of needles inserted,” “needle retention time,” “treatment regimen,” and “precise description of the control intervention” were above 80%. However, the positive reporting rates of items such as “setting and context of treatment” and “practitioner background” were below 20%.

#### 3.4.1. Principle of Acupuncture Treatment

Of the 102 RCTs included, all reported the style of acupuncture treatment (*к* = 1); 84 (82.4%) gave the reasons for providing acupuncture (0.75 < *к* < 1.62); and 27 (26.5%) explained the changes in treatment and selected acupoints according to syndrome differentiation (*к* = 1).

#### 3.4.2. Details of Needling

Among the 102 RCTs included, a vast majority mentioned the number of needles (98.0%, 0.04 < *к* < 1.28), needle stimulation method (92.1%, 0.64 < *к* < 1.02), needle retention time (97.1%, *к* = 1), the number of treatment sessions, and the frequency and duration of treatment sessions (100%, *к* = 1). 62 (60.8%) reported the name of acupoints (0.82 < *к* < 0.98); 36 (35.3%) did not specify whether the acupuncture treatment was given to acupoints on one side or both sides of the body; 4 (3.9%) used nonchannel points without specifying their locations. 71 (69.6%) reported the depth of needle insertion (0.93 < *к* < 1.03). 69 (67.6%) mentioned seeking responses after needle insertion (0.83 < *к* < 0.99). 65 (63.7%) described the needle type (0.85 < *к* < 0.99), and 24 (23.5%) only mentioned the diameter and length of the needle.

#### 3.4.3. Complementary Intervention

Among the 102 RCTs included, 30 (29.4%) administered complementary interventions to the acupuncture group (0.88 < *к* < 1.02); 11 (10.8%) described information including treatment site, instructions to practitioners, and explanations to patients (0.61 < *к* < 0.97).

#### 3.4.4. Practitioner Background

18 (17.6%) described the practitioners' background (0.86 < *к* < 1.02). 1 (1%) only mentioned that acupuncture was given by professional acupuncturists, while the rest did not mention this information.

#### 3.4.5. Control Intervention

The vast majority accurately described the control interventions (*к* = 1), and only 33 (32.4%) described the reasons for control interventions (0.77 < *к* < 1.97).

### 3.5. Agreement between Two Researchers

The two researchers reached substantial (items 3a, 4b, 5b, 5c, 7b, 8b, and 12a), good (items 2a, 6a, 7a, 8a, 9, 10, 11a, 13b, 13c, 14a, 17a, 19, 20, 21, and 22), and prefect (items 1a, 1b, 2b, 3b, 4a, 5, 5a, 5d, 6b, 12b, 13a, 15, 16, 17b, 18, 23, 24, and 25) agreement in all items in Table 1. They also reached substantial (items 2a and 4b), good (items 1b, 2b, 2c, 2d, 2e, 2g, 4a, 5, and 6a), and prefect (items 1a, 1c, 2f, 3a, 3b, and 6b) agreement in all items in Table 2.

## 4. Discussion

This study found that RCTs in acupuncture for PI were varied in reporting quality, with only a few in strict compliance with the CONSORT statement (2017) and STRICTA guidelines.

### 4.1. CONSORT Statement (2017)

In terms of the CONSORT statement (2017), most RCTs had high reporting rates in the introduction, description of trial design, results, and so on. However, the following shortcomings existed: (1) By title, only 15 out of 102 included papers could be identified as RCT. (2) In terms of intervention measures, few RCTs described whether and how to standardize the intervention measures and evaluate the compliance of experimenters and participants with the trial protocol. (3) In terms of sample size, only 14 papers estimated the sample size. (4) In terms of random methods, only a few papers completely reported the generation of sequence, allocation concealment mechanism, implementation, and blinding, while most papers only mentioned the words “random,” “random number table,” or “opaque envelope.” Acupuncture is special in the way that acupuncture practitioners cannot be blinded, so blinding was hardly mentioned in these RCTs. Given that adequate randomization is an effective measure to ensure the authenticity of the results [15], and research confirms that allocation concealment and blinding are important protective measures to reduce the bias of implementation, measurement, and estimation of effects [16] and to ensure the feasibility and repeatability of the research, researchers should describe the random sequence generation, allocation concealment, implementation, and blinding in detail. (5) In terms of results, nearly half of the RCTs reported the number of cases that were dropped out and eliminated from each group, but a small part of them did not explain the reasons. (6) In terms of the implementation of interventions, nearly half of the RCTs only reported the recruitment period or neither the recruitment nor follow-up period; one-third of the RCTs did not use a table to list the baseline data of each group; only five mentioned the estimated effect size and its precision; more than half of the RCTs did not report whether there were harmful or unintended effects in each group. (7) In terms of discussion, more than half of the RCTs did not consider the potential sources of bias, imprecision, and multiple analyses in RCTs; a small number of RCTs did not mention the generalizability of the trial findings; half of the literature only gave the results and did not weigh the benefits and harms of the results or consider other relevant evidence. (8) In terms of other information, although the International Committee of Medical Journal Editors (ICMJE) requires all clinical trials must be registered to improve transparency and accountability [17], few RCTs had clinical trial registration numbers and names of the trial registry; most RCTs did not provide access to a complete trial protocol; more than half of the RCTs did not mention the sources of funding and other support.

### 4.2. STRICTA Guidelines

In terms of STRICTA guidelines, the reporting rates of items 1a, 1b, 2a, 2e, 2f, 3a, 3b, and 6b were above 80%, indicating that most RCTs of acupuncture for PI have paid attention to the contents of these items. However, our study has also found the following shortcomings: (1) In terms of acupuncture details, one-third of the RCTs only mentioned the name of acupoints without the description of whether acupuncture was given to one or both sides. A few RCTs did not report the depth of needle insertion. Some RCTs did not report whether it was necessary to look for acupuncture reaction (Qi arrival) after inserting the needle. A small part of the RCTs did not explain the type of needle, while some mentioned the diameter and length of needles without the name of the manufacturer. The lack of acupuncture details not only reduces the credibility of research conclusions and the objectivity of clinical efficacy but also hinders the promotion and international development of acupuncture therapy [18]. (2) In terms of auxiliary interventions, most of the RCTs did not include the information of treatment and control interventions obtained by patients, such as any wording related to the informed consent and information affecting beliefs and expectations of the treatment. (3) More than 80% of the RCTs did not describe acupuncture practitioners, which could affect the generalizability of the trial results. (4) In terms of control intervention, most RCTs did not explain the reason for selecting the intervention for the control group. The selection of interventions for the control group should be combined with the medical ethics and scientificity of the study, and the reasons should be given [19].

Studies have shown that since CONSORT and STRICTA were firstly introduced to China in 1997 and 2003, respectively, the quality of RCTs report of acupuncture intervention published in Chinese journals has improved over time [12]. The number and quality of RCTs of acupuncture treatment for PI have been greatly improved compared with those before 2010 [20]. Compared with the evaluation of similar acupuncture RCT literature [21, 22], there are still imperfections in the design, implementation of trials, and research reports, while the report on acupuncture details is relatively complete.

### 4.3. Deficiency

This study has its own deficiencies. (1) Only a small number of foreign RCTs in accordance with the inclusion criteria was included, indicating a possible problem of insufficient representation of foreign literature. (2) RCTs were only searched in Chinese and English databases, so RCTs in other languages may be missed.

## 5. Conclusion

In conclusion, the report quality of RCTs of acupuncture for PI needs to be improved. Therefore, it is warranted to advocate the endorsement of the CONSORT statement and STRICTA guidelines for the improvement in the quality of acupuncture RCT reports. For standardized RCT reports and improved quality of acupuncture clinical research, clinical researchers need to learn the basics of clinical trials systematically, and journal editors need to learn and adopt CONSORT statements and STRICTA guidelines.

## Figures and Tables

**Table 1:** Assessment of reporting quality using items from the CONSORT statement (*n* = 102 studies).

Criteria	Item	Description	Number of positive trials	%	Cohen's *к* coefficient	95% CI
Title and abstract	1a	Identification as a randomized trial in the title	15	14.71	1.00	1.00
	1b	Structured abstract including trial design, methods, results, and conclusions	102	100	1.00	1.00
Background and objectives						
Introduction	2a	Scientific background and explanation of rationality	98	96.08	0.85	0.57 to 1.13
	2b	Specific objectives or hypotheses	102	100	1.00	1.00
Trial design						
Methods	3a	Description of trial design	102	100	0.66	0.04 to 1.28
	3b	Important changes to methods after trial commencement (such as eligibility criteria), with reasons	0	0	1.00	1.00
Participants						
	4a	Eligibility criteria for participants	102	100	1.00	1.00
	4b	Settings and locations where the data were collected	100	98.04	0.66	0.04 to 1.28
Interventions						
	5	Interventions for each group with sufficient details to allow replication	101	99.02	1.00	1.00
	5a	Procedure for tailoring the interventions to participants	102	100	1.00	1.00
	5b	Details of whether and how the interventions were standardized	12	11.76	0.77	0.59 to 0.95
	5c	Adherence of researchers to the protocol: whether they adhere to it and how to assess that	5	4.90	0.7	0.42 to 0.98
	5d	Adherence of participants to the intervention: whether they adhere to it and how to assess that	0	0	1.00	1.00
Outcomes						
	6a	Precisely defined prespecified primary and secondary outcome measures	87	85.29	0.84	0.69 to 0.99
	6b	Any changes to trial outcomes after trial commencement with reasons	1	0.98	1.00	1.00
Sample size						
	7a	How sample size was determined	14	13.73	0.87	0.73 to 1.01
	7b	Explanation of interim analyses and suspension principles	17	16.67	0.73	0.57 to 0.89
Sequence generation						
Randomization	8a	Method used to generate the random allocation sequence	69	67.65	0.87	0.77 to 0.97
	8b	Type of randomization with details of any restriction	4	3.92	0.66	0.22 to 1.10
Allocation concealment mechanism						
	9	Description of the method used to implement the random allocation sequence (such as sequentially numbered containers), assuring concealment until interventions were assigned	34	33.33	0.91	0.82 to 1.00
Implementation						
	10	Who generated the random allocation sequence, who enrolled participants, and who assigned intervention to participants	10	9.80	0.95	0.86 to 1.04
Blinding						
	11a	If done, who was blinded and how	23	22.55	0.89	0.79 to 0.99
	11b	If relevant, description of the similarity of interventions	9	8.82	0.8	0.61 to 0.99
	11c	If blinding was not possible, description of attempts to limit bias	7	6.86	0.82	0.58 to 1.06
Statistical methods						
	12a	Statistical methods used to compare groups for primary and secondary outcomes	100	98.04	0.69	0.43 to 0.95
	12b	Methods for additional analyses, such as subgroup analyses and adjusted analyses	2	1.96	1.00	1.00
Participant flow						
Results	13a	For each group, the numbers of participants randomly assigned, received intended treatment, and analyzed for the outcome indicator	102	100	1.00	1.00
	13b	For each group, the number of losses and exclusions after randomization with reasons	33	32.35	0.91	0.84 to 0.98
	13c	For each group, report of the delay from randomization to the initiation of the intervention	4	3.92	0.85	0.57 to 1.13
Recruitment						
	14a	Periods of recruitment and follow-up	55	53.92	0.85	0.75 to 0.95
	14b	Why the trial suspended	0	00	1.00	1.00
Baseline data						
	15	A table showing baseline demographic and clinical characteristics for each group	62	60.78	1.00	1.00
Numbers analyzed						
	16	The number of participants in each group included in each analysis and whether they were analyzed according to the original grouping	102	100	1.00	1.00
Outcomes and estimation						
	17a	The results of primary and secondary outcome indicators, the estimated value of effect size and its accuracy	5	4.90	0.88	0.65 to 1.11
	17b	For dichotomous outcomes, recommendation to provide both absolute and relative effect values	0	0	1.00	1.00
Ancillary analyses						
	18	Results of any other analyses performed, including subgroup analyses and adjusted analyses, distinguishing prespecified analysis from exploratory one	2	1.96	1.00	1.00
Harms						
	19	All important harms or unintended effects in each group	45	44.12	0.96	0.91 to 1.01
Limitations						
Discussion	20	Report of potential source of bias, imprecision, and multiple analyses	47	46.08	0.9	0.82 to 0.98
Generalizability						
	21	Generalizability (external validity, applicability) of the trial findings	65	63.73	0.88	0.79 to 0.97
Interpretation						
	22	Interpretation consistent with results, balancing benefits and harms, and considering other relevant evidence	50	49.02	0.89	0.80 to 0.98
Registration						
Other information	23	Registration number and name of trial registry	12	11.76	1.00	1.00
Protocol						
	24	Where the full-trial protocol can be accessed, if available	36	35.29	1.00	1.00
Funding						
	25	Sources of funding and other support (such as supply of drugs) and role of funders	47	46.08	1.00	1.00

**Table 2:** Assessment of reporting quality of needling details from STRICTA (*n* = 102 studies).

Criteria	Item	Description	Number of positive trials	%	Cohen's *к* coefficient	95% CI
Acupuncture rationale	1a	Style of acupuncture	102	100	1.00	1.00
	1b	Reasons for acupuncture treatment	84	82.35	0.87	0.75 to 1.62
	1c	Explanation of changes was caused by what treatment	27	26.47	1.00	1.00

Needling details	2a	Number of needles insertions per session	100	98.03	0.66	0.04 to 1.28
	2b	Acupoint names (or location in case of nonchannel points) (unilateral or bilateral)	62	60.78	0.90	0.82 to 0.98
	2c	Depth of insertion	71	69.61	0.98	0.93 to 1.03
	2d	Responses sought (arrival of Qi)	69	67.65	0.91	0.83 to 0.99
	2e	Needle stimulation (e.g., manual or electrical)	94	92.16	0.83	0.64 to 1.02
	2f	Needle retention time	99	97.06	1.00	1.00
	2g	Needle type (diameter, length, and manufacturer or material)	65	63.73	0.92	0.85 to 0.99

Treatment regimen	3a	Number of treatment sessions	102	100	1.00	1.00
	3b	Frequency and duration of treatment sessions	102	100	1.00	1.00

Other components of treatment	4a	Complementary interventions for the acupuncture group (e.g., moxibustion, cupping, exercises, and lifestyle advice)	30	29.41	0.95	0.88 to 1.02
	4b	Setting and context of treatment, including instructions to practitioners and information and explanations to patients	11	10.78	0.79	0.61 to 0.97

Practitioner background	5	Description of practitioners	18	17.65	0.94	0.86 to 1.02
Control interventions	6a	Rationale for the control or comparator in the context of the research question, with sources that justify the choice(s)	33	32.35	0.87	0.77 to 0.97
	6b	Precise description of the control or comparator intervention	101	99.02	1.00	1.00

## Data Availability

The dataset can be accessed from the corresponding author upon reasonable request.

## Supplementary Materials

Supplementary Materials S1: search strategies in this paper. S2: evaluation records (CONSORT and STRICTA) by researchers. S3: the information of included papers. S4: the CONSORT checklist. S5: the STRICTA checklist. S6: the total scores for CONSORT and STRICTA.
