# Supplementary material for: Assessment of Reporting Quality in Randomized Controlled Trials of Acupuncture for Primary Insomnia with CONSORT Statement and STRICTA Guidelines
Source: Evid Based Complement Alternat Med. 2022 Feb 17;2022:5157870. doi: 10.1155/2022/5157870 (PMC8872660; doi:10.1155/2022/5157870)
Supplement: Supplementary Materials — S1: search strategies in this paper. S2: evaluation records (CONSORT and STRICTA) by researchers. S3: the information of included papers. S4: the CONSORT checklist. S5: the STRICTA checklist. S6: the total scores for CONSORT and STRICTA. [file 5157870.f1.zip › 5157870.f1/S1 Searching strategy.pdf]

TABLE 1: Searching strategy in CNKI

Search strategy
#1 “Primary insomnia” [Title, Abstract, keywords]
#2 “acupuncture OR body acupuncture OR hand acupuncture OR electroacupuncture OR ear acupuncture OR abdominal acupuncture OR eye acupuncture” [Title, Abstract, keywords]
#3 #1 AND #2

TABLE 2: Searching strategy in VIP

Search strategy
#1 “Primary insomnia” [Title, Abstract, keywords]
#2 “acupuncture OR body acupuncture OR hand acupuncture OR electroacupuncture OR ear acupuncture OR abdominal acupuncture OR eye acupuncture” [Title, Abstract, keywords]
#3 #1 AND #2
#1 “Primary insomnia” [Title, Abstract, keywords]

TABLE 3: Searching strategy in WF

Search strategy
#1 “Primary insomnia” [Title, Abstract, keywords]
#2 “acupuncture OR body acupuncture OR hand acupuncture OR electroacupuncture OR ear acupuncture OR abdominal acupuncture OR eye acupuncture” [Title, Abstract, keywords]
#3 #1 AND #2
#1 “Primary insomnia” [Title, Abstract, keywords]

TABLE 4: Searching strategy in PubMed

Search strategy
#1 “Primary insomnia” [All]
#2 “Acupuncture OR Acupuncture therapy” [All]
#3 “Rct OR Randomized controlled trial ” [All]
#4 #1 AND #2 AND #3

TABLE 5: Searching strategy in EMBASE

Search strategy
#1 “Primary insomnia” [All]
#2 “Acupuncture OR Acupuncture therapy” [All]
#3 “Rct OR Randomized controlled trial ” [All]
#4 #1 AND #2 AND #3

TABLE 6: Searching strategy in Web of Science

Search strategy
#1 AB=(Primary insomnia)
#2 AB=(Acupuncture OR Acupuncture therapy)
#3 AB=(Rct OR Randomized controlled trial)
#4 #1 AND #2 AND #3

TABLE 7: Searching strategy in Cochrane Library

Search strategy
#1 “Primary insomnia” [Title Abstract keywords]
#2 “Acupuncture OR Acupuncture therapy” [Title Abstract keywords]
#3 “Rct OR Randomized controlled trial ” [Title Abstract keywords]
#4 #1 AND #2 AND #3
