# Supplementary material for: Assessment of Reporting Quality in Randomized Controlled Trials of Acupuncture for Primary Insomnia with CONSORT Statement and STRICTA Guidelines
Source: Evid Based Complement Alternat Med. 2022 Feb 17;2022:5157870. doi: 10.1155/2022/5157870 (PMC8872660; doi:10.1155/2022/5157870)
Supplement: Supplementary Materials — S1: search strategies in this paper. S2: evaluation records (CONSORT and STRICTA) by researchers. S3: the information of included papers. S4: the CONSORT checklist. S5: the STRICTA checklist. S6: the total scores for CONSORT and STRICTA. [file 5157870.f1.zip › 5157870.f1/S2 Evaluation record (CONSORT and STRICTA) by researchers.pdf]

No.	year	author	title
1	2021	S. Y. Wan	Clinical Efficacy Observation of Acupuncture Combined with Thumbtack Needle in the Treatment of Primary Insomnia Patients
2	2020	Y. Cao	Effects of Acupuncture Based on Regulating Du Meridian and Calming Mind on Sleep Quality in Patients with Primary Insomnia
3	2020	L. M. Pan	A Randomized Clinical Trial of rTAS in Treating Primary Insomnia
4	2020	Y. Jiang	The clinical efficacy of acupuncture on primary insomnia and the fMRI study for the central mechanism
5	2020	J. Zhao	The clinical efficacy of He's three-pass method in the treatment of subacute insomnia and its effect on the level of neurotransmitter in patients
6	2020	D. F. Li	The clinical efficacy of He's three-pass method in the treatment of subacute insomnia and its effect on the level of neurotransmitter in patients
7	2020	F. J. Liang	Influences of Acupuncture on Serum Brain-gut Peptides and Sleep Quality in Treating Primary Insomnia with Disturbance of Stomach-Qi
8	2020	Q. C. Gao	Clinical Research on the Effects of Acupuncture on Sleep Quality and Cognitive Function for Senile Primary Insomnia
9	2020	Y. K. Wang	The central mechanism study of acupuncture on primary insomnia based on ET and fMRI
10	2020	M. Huang	Clinical study on treating insomnia of liver depression and spleen deficiency with acupuncture of abdomen
11	2020	H. Y. Yan	A Difference Analysis of the Primary Insomnia Treatment by Adjusting the Patients' Wakefulness with Acupuncture and Estazolam
12	2020	Y. Pi	Estimation and Optimization of Commonly-used Acupoint Prescriptions in Acupuncture Treatment of Primary Insomnia Using Spiegel Questionnaire Spiegel
13	2020	Y. Liu	Clinical observation on 36 cases of primary insomnia treated by acupuncture, tuina cooperated with psychological counseling
14	2020	Y. Y. Xu	Clinical Study on Acupuncture of Six Points in Regulating Spirit and Sleep Three-needle Points for Primary Insomnia of Liver Fire Disturbing Heart Type
15	2020	Z. D. Liu	Selection of Acupuncture Schemes and Analysis of Curative Effect for Patients with Different Syndrome Types of Primary Insomnia
16	2019	J. Guo	Clinical Study on the Effect of Acupuncture with Tiaoshen Method on Heart Rate Variability of Primary Insomnia
17	2019	F. Yuan	Clinical study on acupuncture treatment of patients with primary insomnia based on 'Zhoujing Yeming' theory
18	2019	Y. J. Li	Clinical Efficacy Evaluation of Abdominal Acupuncture in Treatment of Chronic Primary Insomnia Heart and Kidney Syndrome and Its Effect on Plasma 5 - HT
19	2019	Y. Kui	Acupuncture for Primary Insomnia: A Systematic Review and A Clinical Study of Balance Acupuncture
20	2019	L. Li	Effect of Acupuncture on the Sleep Quality and Hyperarousal State in Primary Insomnia Patients
21	2019	Z. M. Li	Effects of Soothing Liver, Invigorating Spleen and Tranquilizing Acupuncture on Serum Norepinephrine and Sleep Quality in Patients with Insomnia
22	2019	Z. Z. Tang	Effect of shugan jianpi anshen Acupuncture on 5-hydroxytryptamine and Sleep Quality in Patients with Insomnia
23	2019	H. Y. Ou	Effects of Shugan Jianpi Anshen Acupuncture on serum dopamine and sleep quality in insomnia patients
24	2019	T. Y. Huang	Clinical effects Research and fMRI Research of Neijing "Shu ci " Acupuncture Method in Treatment of Insomnia with Liver-qi Depression and Spleen Deficiency
25	2019	X. H. Li	Clinical observation of the Nazi method of Ziwu Liuzhu acutherapy in the treatment of Cardiac Kindney Non-intercourse of primary insomnia
26	2019	W. Peng	A Study on The Hippocampal Network Regulation Mechanism of Acupuncture of Hewei-Anshen Therapy for Insomnia Patients
27	2019	F. Zhang	Acupuncture Regulating Excessive Arousal of Chronic Insomnia with Deficiency of Heart and Spleen
28	2019	X. P. Yu	Clinical Study on the Effect of Acupuncture on Sleep Quality and Cognitive Function of Elderly Patients with Primary Insomnia
29	2019	W. X. Li	Clinical research on the treatment of primary insomnia with irascibility disturbing heart type by shu-mu points combined with auricular bleeding therapy
30	2018	J. Y. Zhao	Effects of acupuncturing Sanyinjiao (SP6) on PSQI and PSG in patients with primary insomnia
31	2018	J. Qiu	Clinical Observation on Treatment of Primary Insomnia of Liver Stagnation and Spleen Deficiency by Acupuncture with "Regulating Qi Method" and Moxibustion
32	2018	Z. G. Shao	Observation on Therapeutic Effect of Acupuncture Therapy of "Tian Ren Di San Cai" in the Treatment of Primary Insomnia
33	2018	H. H. Gong	The Clinical Study of Seeking Yang in Kan Acupuncturing Method to Treat Imbalance between Heart-yang and kidney-yin Type of Insomnia
34	2018	J. J. Mo	Clinical research of "Sancai acupoints combination" for senile primary insomnia
35	2018	Y. S. Zhu	Tranquilize Mind Acupunctures Treatment of Liver Depression to Fire clinical observation of Primary Insomnia
36	2018	Y. Liang	The Clinical Observation of the treatment of Primary Insomnia (two deficiency of heart and spleen) by Mind-tranquilizing Acupuncture

No.	year	author	title
37	2018	S. J. Wang	Difference Effects of Acupuncturing Single Acupoint or Combinative Acupoints on Quality of Life in Patients with Primary insomnia
38	2017	Y. Shao	Clinical study on acupuncture for primary insomnia
39	2017	W. Z. Shen	Clinical Observation on Abdominal Acupuncture Combined With Plum Blossom Needle in the Treatment of Primary Insomnia
40	2017	H. Q. Li	Treatment of 30 Cases of Primary Insomnia with "Zhou's Tiaoshen Acupuncture Method
41	2017	X. Z. Zhang	Observe the Clinical Efficacy of Shallow Needling for Primary Insomnia and Its Impact on the Neurotransmitter in the Brain
42	2017	Y. Liu	Research on Hyperarousal and Its Associated Nerve Electrophysiology Mechanism in Patients of Primary Insomnia Intervened with Acupuncture
43	2018	J. Y. Zhao	Effects of acupuncturing Sanyinjiao (SP6) on PSQI and PSG in patients with primary insomnia
44	2017	K. Yang	The Effect of Shenmen Acupoints and Sham Acupoints on PSQI and PSG in Patients with Primary Insomnia
45	2017	T. S. Ma	Acupuncture Baihui on PSQI and PSG in Patients with Primary Insomnia
46	2016	Y. K. Gong	Evaluation of the clinical effect of acupuncture in the treatment of primary insomnia from the perspective of the liver
47	2016	B. H. Luo	A comparative study on the clinical efficacy, anxiety and depression scale and sleep index of 121 cases of primary insomnia with three acupuncture methods
48	2016	Y. Pei	The Observation Time Point Injection Adopted Method Combined with the Therapeutic Effect of Electroacupuncture in the Treatment of Primary Insomnia
49	2016	M. X. Zhang	The Effect of Electro Acupuncture Shenmen on PSG and AIS in Patients with Primary Insomnia
50	2016	J. Gao	The Clinical Study of “Ten Acupuncture Point For Sleep Disease” in Treating Primary Insomnia
51	2016	Y. T. Mo	The effect of Sancai Acupoints Combination on ET and PSQI in patients with Primary Insomnia
52	2015	Y. L. Zhu	Effect Observation on Treatment of Primary Insomnia by Head of a Pin Electric Stimulation
53	2015	C. C. Xu	Clinical Observation on Treatment of Primary Insomnia with Ear Acupuncture and Hand Acupuncture
54	2015	Z. Y. Wang	Observation on the Clinical Efficacy of Acupuncture Treating Primary Insomnia from the Perspective of Liver
55	2014	Y. X. Ma	Clinical observation of "old ten needle" treatment of 43 cases of insomnia
56	2014	F. Li	Clinical Curative Effect Observation of Jiaji Electroacupuncture for Patients with Primary Insomnia
57	2014	Y. Y. Liu	Clinical study of Sishenzhen combined Dingshenzhen treatment for primary insomnia
58	2013	D. Y. Wang	Observation on therapeutic effect of scalp acupuncture with cluster needling on primary insomnia
59	2013	J. C. Zhou	Randomized controlled clinical study on treatment of primary insomnia by the head acupoint working in with yuan-source points and collateral-points combination
60	2011	L. N. Huang	Clinical Study on the Efficacy of Scalp Acupuncture for Primary Insomnia
61	2011	D. Su	The Clinical Efficacy of Acupuncture at Yuan-primary point and Luo-connecting Point of the Heart Meridian for the Treatment of Deficiency Insomnia
62	2009	T. He	A meta-analysis of Insomnia-related factors and clinical study of acupuncture
63	2008	J. Guo	Effect of acupuncture on daytime arousal in patients with primary insomnia
64	2008	H. Li	Clinical observation of acupuncture combined with hypnosis in the treatment of primary insomnia
65	2007	Y. B. Xuan	Randomized and controlled study on effect of acupuncture on sleep quality in the patient of primary insomnia
66	2007	X. Wu	A randomized controlled study on the effect of acupuncture with Zhou's Tiaoshen Recipe on sleep quality in patients with primary insomnia
67	2006	L. Luo	Clinical Observations on the Treatment of Insomnia by Herbal Fumigation plus Acupuncture
68	2020	Z. H. Zhao	Effect of acupuncture on sleep quality and excessive arousal in patients with primary insomnia
69	2020	K. X. Huang	Thirty Cases of Chronic Primary Insomnia Treated with Multiple-Needle Superficial Insertion in Combination with the Technique of Regulating the Spine Through Dredging the Governor Vessel
70	2020	Y. X. Lin	Observation on Therapeutic Effect of Acupuncture Combined with Abdominal Breathing on Chronic Insomnia
71	2019	Q. He	A clinical trial of primary insomnia of patients with qi-stagnation constitution by shallow acupuncture combined with ear-acupoint pellet-pressing
72	2018	F. P. Jin	Treatment of 60 cases of primary insomnia with "Tian Di Ren three talents" combined with acupoint acupuncture
73	2013	J. X. Yang	Clinical Effect of Acupuncture and Moxibustion on Insomnia

No.	year	author	title
74	2020	X. H. Wu	Observation on the therapeutic effect of Shu Tiao Balance Acupuncture on insomnia
75	2006	C. H. Zhang	Effects of Electro-puncture Sishencong Points on PSG and PSQI in the Insomnia
76	2015	Y. Y. Wang	Clinical study of insomnia with the treatment of Tongdutiaoshen-Yinqiguiyuan
77	2021	H. Q. Xi	Effect of acupuncture at Tiaoshen acupoints on hyperarousal state in chronic insomnia
78	2021	X. Q. Wang	Effect of electroacupuncture on serum melatonin and dopamine in aged insomnia
79	2021	W. Z. Wu	Effect of Tongdu Tiaoshen acupuncture on serum GABA and CORT levels in patients with chronic insomnia
80	2009	L. S. Huang	The needle-rolling therapy for treatment of non-organic chronic insomnia in 90 cases
81	2008	L. Z. Qi	Observation on the therapeutic effect of neck clustered needling on insomnia
82	2015	X. D. Ji	Expressions of neurotransmitters in patients of insomnia differentiated as liver stagnation transforming into fire treated with acupuncture
83	2009	Y. L. Gong	Clinical observation on therapeutic effect of the pressing plantar reflex area with wooden needle for treatment of patients with insomnia
84	2020	Y. Wang	Effectiveness and cerebral responses of multi-points acupuncture for primary insomnia: a preliminary randomized clinical trial and fMRI study
85	2019	G. Tian-Xian	Effect of "Yinqi Guiyuan" needling on primary insomnia
86	2017	X. Yin	Efficacy and safety of acupuncture treatment on primary insomnia: a randomized controlled trial
87	2016	J. Y. H. Leun	A Clinical Comparison of Verum and Placebo Acupuncture in the Treatment of Primary Insomnia
88	2013	J. Guo	Efficacy of acupuncture for primary insomnia: a randomized controlled clinical trial
89	2014	Y. L. Feng	Clinical effect of Bagua acupuncture combined with Fire Dragon moxibustion in treatment of primary insomnia: a clinical report of 60 cases
90	2012	J. Tu	A comparison between acupuncture versus zolpidem in the treatment of primary insomnia
91	2009	Y. Wing-Fai	Electroacupuncture for primary insomnia: a randomized controlled trial
92	2011	Q. F. Xia	Clinical Research on the Efficacy of "Yin-yang and Qi-blood coordinated cu-point" combined with ear point sticking in Treating Insomnia
93	2019	Y. P. Wang	Yinyang Ruyin acupuncture on refractory insomnia: a randomized controlled trial
94	2020	H. Feng	Effect of acupuncture and estazolam on episodic memory and sleep structure in patients with chronic insomnia disorder: a randomized controlled trial
95	2020	B. Lee	Efficacy and Safety of Electroacupuncture for Insomnia Disorder: A Multicenter, Randomized, Assessor-Blinded, Controlled Trial
96	2015	J. P. Wang	Senile insomnia treated with integrated acupuncture and medication therapy: a randomized controlled trial
97	2008	X. Y. Wang	Abdominal acupuncture for insomnia in women: a randomized controlled clinical trial
98	2010	H. L. Dong	The Randomized clinical trial of the Shaanxi scalp acupuncture treatment of the primary insomnia in randomized, controlled study
99	2017	Y. X. Chen	The study of LingGuiBaFa with respiratory reinforcing and reducing method in the treatment of primary insomnia
100	2009	Q. Y. Feng	Effects of simply embedding therapy of Beishu points on PSQI in patients with primary insomnia
101	2019	F. Xu	Acupoint Catgut Embedding Alleviates Insomnia in Different Chinese Medicine Syndrome Types: A Randomized Controlled Trial
102	2008	M. Sjöling	Auricular acupuncture versus sham acupuncture in the treatment of women who have insomnia

CONSORT (Evaluated by researcher A)

No. \ item	1a	1b	2a	2b	3a	3b	4a	4b	5	5a	5b	5c	5d	6a	6b	7a	7b	8a	8b	9	10	11a	11b	11c	12a	12b	13a	13b	13c	14a
1	0	1	1	1	1	0	1	1	1	1	0	0	0	1	0	0	0	0	0	0	0	0	0	0	1	0	1	0	0	0.5
2	0	1	1	1	1	0	1	1	1	1	0	0	0	1	0	0	0	1	0	1	1	1	1	0	1	0	1	0.5	0	1
3	1	1	1	1	1	0	1	1	1	1	0	0	0	1	0	0	0	1	0	1	0	0	0	0	1	0	1	1	0	1
4	0	1	1	1	1	0	1	1	1	1	0	0	0	1	0	0	1	1	0	0	0	0	1	0	1	1	1	0	0	0.5
5	0	1	1	1	1	0	1	1	1	1	0	0	0	1	0	0	0	1	0	0	0	0	0	0	1	0	1	0	0	0.5
6	0	1	1	1	1	0	1	1	1	1	0	0	0	1	0	0	0	1	0	0	0	0	0	0	1	0	1	0	0	0.5
7	0	1	1	1	1	0	1	1	1	1	1	0	0	1	0	0	0	1	0	0	0	0	0	0	1	0	1	1	0	1
8	0	1	1	1	1	0	1	1	1	1	0	0	0	1	0	0	0	1	0	0	0	0	0	0	1	0	1	1	0	0.5
9	0	1	1	1	1	0	1	1	1	1	0	0	0	1	0	0	1	1	0	0	1	1	0	0	1	0	1	0	1	1
10	0	1	1	1	1	0	1	1	1	1	0	0	0	1	0	1	0	1	0	1	0	1	0	0	1	0	1	1	0	1
11	0	1	0	1	1	0	1	1	0.5	1	0	0	0	1	0	0	0	1	0	0	0	0	0	0	1	0	1	0	0	0.5
12	0	1	1	1	1	0	1	1	1	1	0	0	0	1	0	0	0	0	0	0	0	0	0	0	1	0	1	1	0	1
13	0	1	0	1	1	0	1	1	1	1	0	0	0	1	0	0	0	1	0	0	0	0	0	0	1	0	1	0	0	0.5
14	0	1	1	1	1	0	1	1	1	1	0	0	0	1	0	0	0	0	0	0	0	0	0	0	1	0	1	0	0	1
15	0	1	1	1	1	0	1	1	1	1	0	0	0	1	0	0	0	0	0	0	0	0	0	0	1	0	1	0	0	0.5
16	0	1	1	1	1	0	1	1	1	1	0	0	0	1	0	0	0	0	0	0	0	0	0	0	1	0	1	0.5	0	1
17	0	1	1	1	1	0	1	1	1	1	0	0	0	1	0	0	0	1	0	0	0	0	0	0	1	0	1	1	0	0.5
18	0	1	1	1	1	0	1	1	1	1	0	0	0	1	0	0	0	1	0	0	0	0	0	0	1	0	1	0	0	1
19	0	1	1	1	1	0	1	1	1	1	1	1	0	1	0	1	0	1	1	1	1	1	0	1	1	0	1	1	1	1
20	0	1	1	1	1	0	1	1	1	1	0	0	0	1	0	0	0	1	0	0	0	0	0	0	1	0	1	0	0	0.5
21	0	1	1	1	1	0	1	1	1	1	0	0	0	1	0	0	0	1	0	0	0	0	0	0	1	0	1	0	0	1
22	0	1	1	1	1	0	1	1	1	1	0	0	0	1	0	0	0	0	0	0	0	0	0	0	1	0	1	0	0	1
23	0	1	1	1	1	0	1	1	1	1	0	0	0	1	0	0	0	1	0	0	0	0	0	0	1	0	1	0	0	1
24	0	1	1	1	1	0	1	1	1	1	1	0	0	1	0	1	1	1	0	1	1	1	0	0	1	0	1	0	0	1
25	0	1	1	1	1	0	1	1	1	1	0	0	0	1	0	0	1	1	0	0	0	0	0	0	1	0	1	1	0	0.5
26	0	1	1	1	1	0	1	1	1	1	1	0	0	1	0	1	0	1	0	1	0	1	0	0	1	0	1	1	1	1
27	0	1	1	1	1	0	1	1	1	1	0	0	0	1	0	1	1	1	0	1	0	1	1	1	1	0	1	1	0	1
28	0	1	1	1	1	0	1	1	1	1	0	0	0	1	0	0	0	1	0	0	0	0	0	0	1	0	1	0.5	0	0
29	0	1	1	1	1	0	1	1	1	1	1	1	0	1	0	1	1	1	0	1	1	0	0	0	1	0	1	1	0	0.5
30	0	1	1	1	1	0	1	1	1	1	0	0	0	1	0	0	0	0	0	0	0	0	0	0	1	0	1	1	0	1
31	0	1	1	1	1	0	1	1	1	1	0	0	0	1	0	0	0	1	0	1	0	0	0	0	1	0	1	1	0	0.5
32	0	1	1	1	1	0	1	1	1	1	0	0	0	1	0	0	0	0	0	0	0	0	0	0	1	0	1	0	0	1
33	0	1	1	1	1	0	1	1	1	1	0	0	0	1	0	0	1	0	0	0	0	0	0	0	1	0	1	1	0	1
34	0	1	1	1	1	0	1	1	1	1	0	0	0	1	0	0	1	1	0	1	0	0	0	0	1	0	1	1	0	1

CONSORT (Evaluated by researcher A)

No. \ item	1a	1b	2a	2b	3a	3b	4a	4b	5	5a	5b	5c	5d	6a	6b	7a	7b	8a	8b	9	10	11a	11b	11c	12a	12b	13a	13b	13c	14a
35	0	1	1	1	1	0	1	1	1	1	0	0	0	1	0	0	0	0	0	0	0	0	0	0	1	0	1	1	0	1
36	0	1	1	1	1	0	1	1	1	1	0	0	0	1	0	0	0	1	0	0	0	0	0	0	1	0	1	0	0	1
37	0	1	1	1	1	0	1	1	1	1	0	0	0	1	0	0	0	1	0	0	0	0	0	0	1	0	1	1	0	1
38	0	1	1	1	1	0	1	1	1	1	0	0	0	1	0	0	0	0	0	0	0	0	0	0	1	0	1	0	0	0.5
39	0	1	1	1	1	0	1	1	1	1	0	0	0	0.5	0	0	0	0	0	0	0	0	0	0	1	0	1	0	0	0.5
40	0	1	1	1	1	0	1	1	1	1	0	0	0	1	0	0	0	0	0	0	0	1	0	0	1	0	1	0	0	1
41	0	1	1	1	1	0	1	1	1	1	1	1	0	1	0	1	1	1	0	1	0	1	0	0	1	1	1	1	0	1
42	0	1	1	1	1	0	1	1	1	1	0	0	0	1	0	0	0	1	0	0	0	0	0	0	1	0	1	0.5	0	0.5
43	0	1	1	1	1	0	1	1	1	1	0	0	0	1	0	0	0	0	0	0	0	0	0	0	1	0	1	1	0	1
44	0	1	1	1	1	0	1	1	1	1	0	0	0	1	0	0	0	1	0	1	0	0	0	0	1	0	1	0.5	0	1
45	0	1	1	1	1	0	1	1	1	1	0	0	0	1	0	0	0	1	0	1	0	0	0	0	1	0	1	1	0	1
46	0	1	0	1	1	0	1	1	1	1	0	0	0	0.5	0	0	0	0	0	0	0	0	0	0	0.5	0	1	0	0	0.5
47	0	1	1	1	1	0	1	1	1	1	0	0	0	0.5	0	0	1	1	1	1	0	0	0	0	0.5	0	1	1	0	1
48	0	1	1	1	1	0	1	1	1	1	1	0	0	1	0	0	0	1	0	0	0.5	0	0	1	1	0	1	0.5	0	0.5
49	0	1	1	1	1	0	1	1	1	1	0	0	0	1	0	0	0	1	0	1	0	0	0	0	1	0	1	0	0	0
50	0	1	1	1	1	0	1	1	1	1	0	0	0	1	0	0	0	0	0	0	0	0	0	0	1	0	1	0	0	1
51	0	1	1	1	1	0	1	1	1	1	0	0	0	1	0	0	0	1	0	0	0	0	0	0	1	0	1	1	0	1
52	0	1	1	1	1	0	1	1	1	1	0	0	0	1	0	0	0	0	0	0	0	0	0	0	1	0	1	0	0	0.5
53	0	1	1	1	1	0	1	1	1	1	0	0	0	0.5	0	0	0	1	0	0	0	0	0	0	1	0	1	0	0	0
54	0	1	1	1	1	0	1	1	1	1	0	0	0	1	0	0	1	1	0	0	0	0	0	0	1	0	1	0	0	0.5
55	0	1	1	1	1	0	1	1	1	1	0	0	0	1	0	0	0	0	0	0	0	0	0	0	1	0	1	0	0	0.5
56	0	1	1	1	1	0	1	1	1	1	0	0	0	1	0	0	0	1	0	0	0	0	0	1	1	0	1	0	0	1
57	0	1	1	1	1	0	1	1	1	1	0	0	0	1	0	0	1	1	0	0	0	0	0	0	1	0	1	0	0	0.5
58	0	1	1	1	1	0	1	1	1	1	0	0	0	1	0	0	0	0	0	0	0	0	0	0	1	0	1	0	0	0
59	1	1	1	1	1	0	1	1	1	1	1	0	0	1	0	0	1	1	0	1	0	1	0	1	1	0	1	1	0	1
60	0	1	1	1	1	0	1	1	1	1	0	0	0	1	0	0	0	1	0	0	0	0	0	0	1	0	1	0	0	0.5
61	0	1	1	1	1	0	1	0	1	1	0	0	0	1	0	0	0	0	0	0	0	0	0	0	1	0	1	0	0	0
62	0	1	1	1	1	0	1	1	1	1	0	0	0	1	0	0	0	1	1	1	0	0	0	0	1	0	1	0	0	1
63	0	1	1	1	1	0	1	1	1	1	0	0	0	1	0	0	0	1	0	1	0	0	0	0	1	0	1	0	0	1
64	0	1	0	1	1	0	1	1	1	1	0	0	0	0.5	0	0	0	0	0	0	0	0	0	0	0	0	1	0	0	0.5
65	1	1	1	1	0	0	1	1	1	1	0	0	0	0.5	0	0	0	1	0	1	0	0	0	0	1	0	1	0	0	1
66	1	1	1	1	1	0	1	1	1	1	0	0	0	0.5	0	1	0	1	0	1	0	0	0	0	1	0	1	0	0	1
67	0	1	1	1	1	0	1	1	1	1	0	0	0	0.5	0	0	0	0	0	0	0	0	0	0	0	0	1	0	0	0.5
68	0	1	1	1	1	0	1	1	1	1	0	0	0	1	0	0	0	1	0	0	0	0	0	0	1	0	1	0	0	0.5

CONSORT (Evaluated by researcher A)

No. \ item	1a	1b	2a	2b	3a	3b	4a	4b	5	5a	5b	5c	5d	6a	6b	7a	7b	8a	8b	9	10	11a	11b	11c	12a	12b	13a	13b	13c	14a
69	0	1	1	1	1	0	1	1	1	1	0	0	0	1	0	0	0	1	0	0	0	0	0	0	1	0	1	0	0	0.5
70	0	1	1	1	1	0	1	1	1	1	0	0	0	1	0	0	0	1	0	0	0	0	0	0	1	0	1	0	0	1
71	0	1	1	1	1	0	1	1	1	1	0	0	0	1	0	0	0	1	0	0	0	0	0	0	1	0	1	0	0	1
72	0	1	1	1	1	0	1	1	1	1	0	0	0	1	0	0	0	0	0	0	0	0	0	0	1	0	1	0	0	1
73	0	1	1	1	1	0	1	1	1	1	0	0	0	0.5	0	0	0	1	0	0	0	0	0	0	1	0	1	0	0	0.5
74	0	1	1	1	1	0	1	1	1	1	0	0	0	1	0	0	0	0	0	0	0	0	0	0	1	0	1	0	0	1
75	0	1	1	1	1	0	1	1	1	1	0	0	0	1	0	0	0	0	0	0	0	0	0	0	1	0	1	0	0	1
76	0	1	1	1	1	0	1	1	1	1	0	0	0	0.5	0	0	1	1	0	0	0	0	0	0	1	0	1	0	0	0.5
77	0	1	1	1	1	0	1	1	1	1	0	0	0	1	0	0	0	1	0	1	0	1	0	0	1	0	1	1	0	0.5
78	0	1	1	1	1	0	1	1	1	1	0	0	0	1	0	1	0	1	0	1	0	1	0	0	1	0	1	1	0	0.5
79	0	1	1	1	1	0	1	1	1	1	0	0	0	1	0	0	0	0	0	1	0	1	0	0	1	0	1	1	0	0.5
80	0	1	1	1	1	0	1	1	1	1	0	0	0	1	0	0	0	1	0	1	0	0	0	0	1	0	1	0	0	1
81	0	1	1	1	1	0	1	1	1	1	0	0	0	0.5	0	0	0	0	0	0	0	0	0	0	1	0	1	0	0	0.5
82	0	1	1	1	1	0	1	1	1	1	0	0	0	1	0	0	0	1	0	0	0	0	0	0	1	0	1	0	0	0.5
83	0	1	1	1	1	0	1	1	1	1	0	0	0	1	0	0	0	0	0	0	0	0	0	0	1	0	1	0	0	1
84	1	1	1	1	1	0	1	1	1	1	1	0	0	1	0	0	0	1	0	0	1	1	1	0	1	0	1	0	0	1
85	0	1	1	1	1	0	1	1	1	1	0	0	0	1	0	0	0	1	0	0	0	0	0	0	1	0	1	1	0	0.5
86	1	1	1	1	1	0	1	1	1	1	1	1	0	1	0	1	0	1	0	1	1	1	1	1	1	0	1	1	0	1
87	0	1	1	1	1	0	1	1	1	1	0	0	0	0.5	0	0	1	0	0	0	0	0.5	0	0	0.5	0	1	0	0	0.5
88	1	1	1	1	1	0	1	1	1	1	1	0	0	1	0	1	0	1	0	1	0	1	1	1	1	0	1	0.5	0	0.5
89	0	1	1	1	1	0	1	1	1	1	0	0	0	1	0	0	0	1	0	0	0	0	0	0	1	0	1	0	0	1
90	0	1	1	1	1	0	1	1	1	1	0	0	0	0.5	0	0	0	0	0	0	0	0	0	0	1	0	1	1	0	1
91	1	1	1	1	1	0	1	0	1	1	0	0	0	1	0	1	0	1	0	1	1	1	0	0	1	0	1	0.5	1	1
92	0	1	1	1	1	0	1	1	1	1	0	0	0	0.5	0	0	0	0	0	0	0	0	0	0	1	0	1	0	0	1
93	1	1	1	1	1	0	1	1	1	1	0	0	0	1	0	0	1	1	0	0	0	0	0	0	1	0	1	0	0	0.5
94	1	1	1	1	1	0	1	1	1	1	0	0	0	1	0	0	0	1	0	1	0	0	0	0	1	0	1	1	0	0.5
95	1	1	1	1	1	0	1	1	1	1	0	0	0	1	0	1	0	1	0	1	0	1	1	0	1	0	1	1	0	1
96	1	1	1	1	1	0	1	1	1	1	0	0	0	1	1	0	0	1	0	0	0	0	0	0	1	0	1	0	0	1
97	1	1	1	1	1	0	1	1	1	1	0	0	0	1	0	0	0	1	0	1	0	1	1	0	1	0	1	0	0	0.5
98	1	1	1	1	1	0	1	1	1	1	0	0	0	1	0	0	0	1	0	1	0	0	0	0	1	0	1	1	0	1
99	0	1	1	1	1	0	1	1	1	1	0	0	0	1	0	0	0	1	0	1	1	1	0	0	1	0	1	1	0	0.5
100	0	1	1	1	1	0	1	1	1	1	0	0	0	0.5	0	0	1	0	0	0	0	0	0	0	0.5	0	1	0	0	0
101	1	1	1	1	1	0	1	1	1	1	0	0	0	1	0	1	0	1	1	1	1	0	0	0	1	0	1	0.5	0	1
102	0	1	1	1	1	0	1	1	1	1	1	1	0	1	0	1	0	1	0	1	0	1	1	0	1	0	1	0.5	0	1

CONSORT (Evaluated by researcher A)

No. \ item	14b	15	16	17a	17b	18	19	20	21	22	23	24	25
1	0	0.5	1	0	0	0	0	0	1	0.5	0	1	0
2	0	1	1	1	0	0	0	0	1	0.5	0	1	1
3	0	1	1	0	0	0	1	1	1	0.5	0	0	1
4	0	0.5	1	0	0	1	0	1	1	1	1	0	1
5	0	0.5	1	0	0	0	0	1	1	1	0	0	0
6	0	0.5	1	0	0	0	0	0	1	1	0	0	0
7	0	0.5	1	0	0	0	1	1	1	1	0	1	1
8	0	1	1	0	0	0	0	1	0	1	0	0	0
9	0	1	1	0	0	0	0	0	1	0.5	1	0	0
10	0	1	1	0	0	0	1	1	1	1	0	0	0
11	0	0.5	1	0	0	0	0	0	1	0.5	0	1	1
12	0	1	1	0	0	0	0	0	0	0	0	1	1
13	0	0.5	1	0	0	0	0	0	1	0.5	0	0	0
14	0	0.5	1	0	0	0	0	0	1	0.5	0	0	0
15	0	0.5	1	0	0	0	1	0	0	0.5	0	0	0
16	0	0.5	1	0	0	0	1	1	0	1	0	1	1
17	0	1	1	0	0	0	1	0	1	0.5	0	1	1
18	0	0	1	0	0	0	0	0	0	0.5	0	0	1
19	0	1	1	0	0	0	1	1	1	1	0	0	0
20	0	1	1	0	0	0	0	0	0	0.5	0	1	0
21	0	1	1	0	0	0	1	1	1	1	0	0	1
22	0	1	1	0	0	0	1	1	1	1	0	0	0
23	0	1	1	0	0	0	1	1	1	1	0	0	1
24	0	1	1	0	0	0	0	1	0	1	0	0	0
25	0	1	1	0	0	0	1	1	1	1	0	0	0
26	0	1	1	0	0	0	0	1	0	1	1	0	1
27	0	1	1	0	0	1	0	1	1	1	0	0	0
28	0	0.5	1	0	0	0	0	0	0	0.5	0	1	0
29	0	1	1	0	0	0	1	1	1	1	0	0	0
30	0	1	1	0	0	0	0	0	1	0.5	0	1	1
31	0	0.5	1	0	0	0	0	0	1	0.5	0	0	1
32	0	0.5	1	0	0	0	0	0	0	0.5	0	0	0
33	0	1	1	0	0	0	1	1	1	1	0	0	0
34	0	1	1	0	0	0	1	0	1	1	0	0	0

CONSORT (Evaluated by researcher A)

No. \ item	14b	15	16	17a	17b	18	19	20	21	22	23	24	25
35	0	1	1	0	0	0	1	1	1	1	0	0	1
36	0	1	1	0	0	0	1	1	1	1	0	0	0
37	0	1	1	0	0	0	0	1	0	1	0	0	1
38	0	1	1	0	0	0	1	0	1	0.5	0	1	0
39	0	0.5	1	0	0	0	0	0	0	0.5	0	0	0
40	0	0.5	1	0	0	0	0	1	0	1	0	1	0
41	0	1	1	0	0	0	1	1	1	1	0	0	0
42	0	1	1	0	0	0	0	0	0	1	0	0	1
43	0	1	1	0	0	0	0	1	1	1	0	1	1
44	0	1	1	0	0	0	1	1	1	1	0	0	1
45	0	1	1	0	0	0	1	1	0	1	0	0	1
46	0	0.5	1	0	0	0	0	0	1	0.5	0	0	0
47	0	0.5	1	0	0	0	0	0	1	0.5	0	0	1
48	0	1	1	0	0	0	0	0	1	0.5	0	0	0
49	0	1	1	0	0	0	1	1	1	1	0	0	1
50	0	1	1	0	0	0	0	0	1	1	0	0	0
51	0	1	1	0	0	0	1	1	0	1	0	0	1
52	0	1	1	0	0	0	0	0	1	0.5	0	0	0
53	0	0.5	1	0	0	0	1	0	0	0	0	0	1
54	0	0.5	1	0	0	0	0	0	1	0.5	0	0	0
55	0	0.5	1	0	0	0	0	0	1	0.5	0	0	0
56	0	1	1	0	0	0	0	1	0	1	0	0	0
57	0	1	1	0	0	0	0	1	1	1	0	0	0
58	0	0.5	1	0	0	0	0	0	1	0.5	0	0	0
59	0	1	1	0	0	0	1	0	1	0.5	0	0	0
60	0	0.5	1	0	0	0	0	0	1	0.5	0	0	1
61	0	0.5	1	0	0	0	1	0	0	0.5	0	0	0
62	0	0.5	1	0	0	0	0	1	1	1	0	0	0
63	0	0.5	1	0	0	0	0	0	1	0.5	0	0	1
64	0	0.5	1	0	0	0	1	0	1	0.5	0	0	1
65	0	0.5	1	0	0	0	0	0	0	0.5	0	1	0
66	0	1	1	0	0	0	0	1	0	1	0	0	0
67	0	0	1	0	0	0	0	0	0	0.5	0	0	0
68	0	0.5	1	0	0	0	0	0	1	0.5	0	0	0

CONSORT (Evaluated by researcher A)

No. \ item	14b	15	16	17a	17b	18	19	20	21	22	23	24	25
69	0	0.5	1	0	0	0	1	0	1	0.5	0	1	1
70	0	1	1	0	0	0	0	0	1	0.5	0	0	0
71	0	0.5	1	1	0	0	1	0	1	0.5	0	1	0
72	0	0.5	1	0	0	0	0	0	1	0.5	0	0	0
73	0	1	1	0	0	0	0	0	1	0.5	0	1	1
74	0	0.5	1	0	0	0	0	0	1	0.5	0	1	1
75	0	1	1	0	0	0	0	0	0	0.5	0	0	0
76	0	1	1	0	0	0	0	1	1	1	0	0	0
77	0	1	1	0	0	0	1	1	0	1	1	1	1
78	0	1	1	0	0	0	0	0	0	0.5	1	1	1
79	0	1	1	0	0	0	0	1	0	1	1	1	1
80	0	0.5	1	0	0	0	1	0	0	0.5	0	0	0
81	0	1	1	0	0	0	1	1	0	1	0	1	1
82	0	0.5	1	0	0	0	0	0	0	0.5	0	1	1
83	0	1	1	0	0	0	1	0	1	0.5	0	1	0
84	0	1	1	0	0	0	1	1	1	1	1	1	1
85	0	0.5	1	0	0	0	1	0	1	0.5	0	1	1
86	0	1	1	0	0	0	1	1	1	1	1	1	1
87	0	1	1	0	0	0	1	1	1	1	0	0	0
88	0	1	1	1	0	0	1	1	0	1	1	1	1
89	0	0.5	1	0	0	0	0	0	1	0.5	0	1	1
90	0	1	1	0	0	0	1	1	1	1	0	1	0
91	0	1	1	0	0	0	1	1	0	1	1	0	0
92	0	0.5	1	0	0	0	0	0	1	0.5	0	0	1
93	0	1	1	0	0	0	0	0	0	0.5	0	1	1
94	0	1	1	0	0	0	0	0	0	0.5	1	1	1
95	0	1	1	1	0	0	1	1	0	1	1	1	1
96	0	1	1	0	0	0	1	0	1	0.5	0	1	1
97	0	1	1	1	0	0	1	1	0	1	0	1	0
98	0	1	1	0	0	0	1	1	1	1	0	0	0
99	0	1	1	0	0	0	1	1	0	1	0	0	0
100	0	1	1	0	0	0	0	1	1	1	0	0	0
101	0	0	1	0	0	0	1	0	1	0.5	0	1	1
102	0	1	1	0	0	0	0	1	0	1	0	0	1

STRICTA (Evaluated by researcher A)

No.	item 1a	1b	1c	2a	2b	2c	2d	2e	2f	2g	3a	3b	4a	4b	5	6a	6b
1	1	1	0	1	1	0	0	0	1	0	1	1	1	0	1	0	1
2	1	1	0	1	0.5	1	1	1	1	0	1	1	0	0	0	0	1
3	1	1	0	1	1	1	1	1	1	1	1	1	0	0	0	0	1
4	1	1	0	1	0.5	1	0	1	1	1	1	1	0	0	1	1	1
5	1	1	0	1	1	1	1	1	1	0.5	1	1	1	0	0	0	1
6	1	1	0	1	1	1	1	1	1	0.5	1	1	1	0	0	0	1
7	1	1	0	1	1	1	1	1	1	1	1	1	1	0	1	0	1
8	1	1	0	1	1	1	1	1	1	1	1	1	0	0	0	0	1
9	1	1	0	1	0.5	1	1	1	1	1	1	1	1	0	1	0	1
10	1	1	0	1	1	0	1	1	1	1	1	1	1	0	0	0	1
11	1	1	1	1	0.5	0	0	0	0	0	1	1	0	1	0	0	1
12	1	1	0	1	0.5	1	0	1	1	1	1	1	0	0	0	0	1
13	1	0	1	1	1	0	1	1	1	1	1	1	1	1	0	0	1
14	1	1	0	1	0.5	1	1	1	1	0.5	1	1	0	0	0	0	1
15	1	1	1	1	0.5	0	0	1	1	0	1	1	1	0	0	0	1
16	1	1	0	1	0.5	0	1	1	1	1	1	1	0	0	0	1	1
17	1	1	0	1	1	0	1	1	1	1	1	1	0	0	0	1	1
18	1	1	0	1	1	0.5	0	1	1	0.5	1	1	0	0	0	0	1
19	1	1	0	1	0.5	1	1	1	1	1	1	1	0	1	0	1	1
20	1	1	0	1	0.5	1	1	1	1	0.5	1	1	0	0	0	0	1
21	1	1	0	1	1	1	1	1	1	1	1	1	0	0	0	1	1
22	1	1	0	1	1	1	1	1	1	1	1	1	0	0	0	1	1
23	1	1	0	1	1	1	1	1	1	1	1	1	0	0	0	1	1
24	1	1	0	1	1	1	1	1	1	1	1	1	0	0	0	1	1
25	1	1	0	1	1	0	1	1	1	1	1	1	0	0	0	0	1
26	1	1	0	1	0.5	1	1	1	1	1	1	1	0	1	1	1	1
27	1	1	0	1	1	1	1	1	1	1	1	1	0	0	1	1	1
28	1	1	0	1	1	1	1	1	1	1	1	1	0	0	0	0	0
29	1	1	0	1	1	1	1	1	1	0.5	1	1	1	0	1	0	1
30	1	1	0	1	1	1	1	1	1	1	1	1	0	0	0	0	1
31	1	1	1	1	1	1	1	1	1	1	1	1	1	0	0	0	1
32	1	1	1	1	1	1	0	1	1	0.5	1	1	1	0	0	0	1
33	1	1	0	1	0.5	0	0	1	1	1	1	1	0	0	0	0	1
34	1	1	1	1	1	1	1	1	1	1	1	1	1	0	0	0	1
35	1	1	0	1	1	1	1	1	1	1	1	1	0	0	0	1	1

STRICTA (Evaluated by researcher A)

No. \ item	1a	1b	1c	2a	2b	2c	2d	2e	2f	2g	3a	3b	4a	4b	5	6a	6b
36	1	1	0	1	1	0.5	0	1	1	1	1	1	0	0	0	1	1
37	1	1	0	1	1	1	1	1	1	1	1	1	0	0	0	1	1
38	1	1	1	1	0	0	0	1	1	0.5	1	1	0	0	0	0	1
39	1	1	1	1	1	1	0	1	1	1	1	1	1	0	0	0	1
40	1	1	0	1	1	0	1	1	1	1	1	1	0	0	0	1	1
41	1	1	0	1	1	1	1	1	1	0.5	1	1	0	0	1	0	1
42	1	0	1	1	0	1	1	1	1	1	1	1	0	0	0	0	1
43	1	1	0	1	1	1	0	1	1	1	1	1	0	0	0	0	1
44	1	1	0	1	1	1	1	1	1	1	1	1	0	0	0	1	1
45	1	1	0	1	1	1	0	1	1	1	1	1	0	0	0	0	1
46	1	0	1	1	0.5	0	0	1	1	0	1	1	0	0	0	0	1
47	1	1	1	1	0.5	0	0	1	1	0.5	1	1	0	0	0	0	1
48	1	1	1	1	0.5	1	1	1	1	1	1	1	0	0	0	1	1
49	1	1	0	1	1	1	1	1	1	1	1	1	0	0	0	0	1
50	1	1	0	1	1	1	1	1	1	1	1	1	0	0	0	0	1
51	1	1	0	1	1	1	0	1	1	1	1	1	0	0	0	0	1
52	1	0	1	1	1	1	1	1	1	0	1	1	0	0	0	0	1
53	1	0	0	1	1	0	0	1	0	0.5	1	1	0	0	0	1	1
54	1	1	1	1	0.5	0	0	1	1	0	1	1	0	0	0	1	1
55	1	0	0	1	0.5	0	1	0	1	1	1	1	0	0	0	0	1
56	1	1	0	1	1	1	1	1	1	0.5	1	1	0	0	0	1	1
57	1	1	0	1	1	0	1	1	1	1	1	1	0	0	0	0	1
58	1	0	0	1	0.5	0	1	1	1	0	1	1	0	0	0	0	1
59	1	1	1	1	0.5	1	1	1	1	1	1	1	0	0	0	0	1
60	1	0	0	1	0.5	1	1	1	1	1	1	1	0	0	0	0	1
61	1	1	1	1	0.5	0	1	1	1	0.5	1	1	0	0	0	0	1
62	1	1	0	1	1	1	1	1	1	0.5	1	1	0	0	0	0	1
63	1	1	0	1	0.5	0	0	1	1	1	1	1	0	0	0	0	1
64	1	0	1	1	1	1	0	1	1	0.5	1	1	1	0	0	0	1
65	1	1	0	1	0.5	1	0	1	1	1	1	1	0	0	0	0	1
66	1	1	0	1	1	0	0	1	1	1	1	1	0	0	0	0	1
67	1	0	0	1	1	0	1	1	1	0	1	1	1	0	0	0	1
68	1	0	0	1	1	1	1	1	1	0.5	1	1	0	0	0	0	1
69	1	0	1	1	1	0	0	0	1	1	1	1	1	0	0	0	1
70	1	0	0	1	1	1	0	1	1	1	1	1	1	0	0	0	1

STRICTA (Evaluated by researcher A)

No.	item 1a	1b	1c	2a	2b	2c	2d	2e	2f	2g	3a	3b	4a	4b	5	6a	6b
71	1	1	0	1	1	1	0	1	1	1	1	1	1	0	0	0	1
72	1	1	0	1	1	1	0	1	1	0.5	1	1	0	0	0	0	1
73	1	1	0	1	1	1	0	1	1	1	1	1	1	0	0	0	1
74	1	1	1	1	0.5	1	0	1	0	0	1	1	1	0	0	0	1
75	1	1	0	1	1	1	1	1	1	1	1	1	0	0	0	1	1
76	1	1	0	1	1	0	1	1	1	1	1	1	0	0	0	0	1
77	1	1	0	1	0.5	1	1	1	1	1	1	1	1	0	1	0	1
78	1	1	0	1	0.5	1	1	1	1	0.5	1	1	1	0	1	0	1
79	1	1	0	1	0.5	1	1	0	1	0.5	1	1	1	0	1	0	1
80	1	1	0	1	0.5	0	1	1	1	0.5	1	1	1	0	0	0	1
81	1	1	0	1	1	1	1	1	1	0.5	1	1	0	0	0	1	1
82	1	1	0	1	0.5	1	1	1	1	1	1	1	0	0	0	0	1
83	1	0	1	1	1	1	1	1	1	1	1	1	0	0	0	0	1
84	1	1	0	1	1	1	1	1	1	1	1	1	0	1	1	1	1
85	1	1	1	1	0	0	1	1	1	1	1	1	0	0	0	0	1
86	1	1	0	1	1	1	1	1	1	0	1	1	1	0	1	1	1
87	1	1	0	1	1	1	0	1	1	1	1	1	0	0	0	1	1
88	1	1	0	1	0.5	1	1	1	1	1	1	1	1	1	1	1	1
89	1	0	1	1	1	1	0	0	1	0	1	1	1	0	0	0	1
90	1	1	0	1	1	1	1	1	1	0	1	1	0	1	1	0	1
91	1	1	0	1	1	0	1	1	1	1	1	1	0	1	0	1	1
92	1	0	1	1	0.5	1	1	1	1	1	1	1	1	0	0	0	1
93	1	0	1	1	0.5	1	0	1	1	0.5	1	1	0	0	0	1	1
94	1	0	0	1	0.5	1	1	1	1	0.5	1	1	0	0	1	1	1
95	1	1	0	1	1	1	1	1	1	1	1	1	0	1	1	1	1
96	1	1	1	0	0.5	1	1	1	1	1	1	1	0	0	0	0	1
97	1	1	0	1	1	1	0	0	1	0.5	1	1	1	0	0	1	1
98	1	1	0	1	0	1	1	1	1	1	1	1	0	0	0	1	1
99	1	1	1	0	0.5	0	1	1	1	1	1	1	0	0	0	1	1
100	1	1	0	1	1	1	1	1	1	1	1	1	0	0	0	1	1
101	1	1	1	1	0.5	1	1	1	1	1	1	1	0	1	1	0	1
102	1	1	0	1	1	0	0	0	1	1	1	1	0	1	1	0	1

CONSORT (Evaluated by researcher B)

item	1a	1b	2a	2b	3a	3b	4a	4b	5	5a	5b	5c	5d	6a	6b	7a	7b	8a	8b	9	10	11a	11b	11c	12a	12b	13a	13b	13c	14a	14b
No.																															
1	0	1	1	1	1	0	1	1	1	1	0	0	0	1	0	0	0	0	0	0	0	0	0	0	1	0	1	0	0	1	0
2	0	1	1	1	1	0	1	1	1	1	0	0	0	1	0	0	0	1	0	1	1	1	1	0	1	0	1	0.5	0	1	0
3	1	1	1	1	1	0	1	1	1	1	0	0	0	1	0	0	0	1	0	1	0	0	0	0	1	0	1	1	0	1	0
4	0	1	1	1	1	0	1	1	1	1	0	0	0	1	0	0	1	1	0	0	0	0	1	0	1	1	1	0	0	1	0
5	0	1	1	1	1	0	1	1	1	1	0	0	0	1	0	0	0	1	0	0	0	0	0	0	1	0	1	0.5	0	1	0
6	0	1	1	1	1	0	1	1	1	1	0	0	0	1	0	0	0	1	0	0	0	0	0	0	1	0	1	0	0	1	0
7	0	1	1	1	1	0	1	1	1	1	1	1	0	1	0	0	0	1	0	0	0	0	0	0	1	0	1	1	0	1	0
8	0	1	1	1	1	0	1	1	1	1	0	0	0	1	0	0	0	1	0	0	0	0	0	0	1	0	1	1	0	1	0
9	0	1	1	1	1	0	1	1	1	1	0	0	0	1	0	0	1	0	0	0	1	1	0	0	1	0	1	0	1	1	0
10	0	1	1	1	1	0	1	1	1	1	0	0	0	1	0	1	0	1	0	1	0	1	0	0	1	0	1	1	0	1	0
11	0	1	0	1	1	0	1	1	0.5	1	1	1	0	1	0	0	1	1	0	0	0	0	0	0	1	0	1	0	0	0.5	0
12	0	1	1	1	1	0	1	1	1	1	0	0	0	1	0	0	0	0	0	0	0	0	0	0	1	0	1	1	0	1	0
13	0	1	1	1	1	0	1	1	1	1	0	0	0	1	0	0	0	1	0	0	0	0	0	0	1	0	1	0	0	0.5	0
14	0	1	1	1	1	0	1	1	1	1	0	0	0	1	0	0	0	0	0	0	0	0	0	0	1	0	1	0	0	1	0
15	0	1	1	1	1	0	1	1	1	1	0	0	0	1	0	0	0	0	0	0	0	0	0	0	1	0	1	0	0	0.5	0
16	0	1	1	1	1	0	1	1	1	1	0	0	0	1	0	0	0	0	0	0	0	0	0	0	1	0	1	0.5	0	1	0
17	0	1	1	1	1	0	1	1	1	1	0	0	0	1	0	0	0	1	0	0	0	0	0	0	1	0	1	1	0	0.5	0
18	0	1	1	1	1	0	1	1	1	1	0	0	0	1	0	0	0	1	0	0	0	0	0	0	1	0	1	0	0	1	0
19	0	1	1	1	1	0	1	1	1	1	1	1	0	1	0	0	0	1	0	1	1	1	0	1	1	0	1	1	1	1	0
20	0	1	1	1	1	0	1	1	1	1	0	0	0	1	0	0	0	1	0	0	0	0	0	0	1	0	1	0	0	0.5	0
21	0	1	1	1	1	0	1	1	1	1	0	0	0	1	0	0	1	1	0	0	0	0	0	0	1	0	1	0	0	1	0
22	0	1	1	1	1	0	1	1	1	1	0	0	0	1	0	0	1	0	0	0	0	0	0	0	1	0	1	0	0	1	0
23	0	1	1	1	1	0	1	1	1	1	1	0	0	1	0	0	1	1	0	0	0	0	0	0	1	0	1	0	0	1	0
24	0	1	1	1	1	0	1	1	1	1	1	0	0	1	0	1	1	1	0	1	1	1	0	0	1	0	1	0	0	1	0
25	0	1	1	1	1	0	1	1	1	1	0	0	0	1	0	0	1	1	0	0	0	0	0	0	1	0	1	1	0	0.5	0
26	0	1	1	1	1	0	1	1	1	1	1	0	0	1	0	1	1	1	0	1	0	1	1	0	1	0	1	1	1	1	0
27	0	1	1	1	1	0	1	1	1	1	0	0	0	1	0	0	1	1	0	1	0	1	1	1	1	0	1	1	0	1	0
28	0	1	1	1	1	0	1	1	1	1	0	0	0	1	0	0	0	1	0	0	0	0	0	0	1	0	1	0.5	0	0	0
29	0	1	1	1	1	0	1	1	1	1	1	1	0	1	0	1	1	1	0	1	1	0	0	0	1	0	1	1	0	0.5	0
30	0	1	1	1	1	0	1	1	1	1	0	0	0	1	0	0	0	0	0	0	0	0	1	0	1	0	1	1	0	1	0
31	0	1	1	1	1	0	1	1	1	1	0	0	0	1	0	0	0	1	0	1	0	0	0	0	1	0	1	1	0	0.5	0
32	0	1	1	1	1	0	1	1	1	1	0	0	0	1	0	0	0	0	0	0	0	0	0	0	1	0	1	0	0	1	0
33	0	1	1	1	1	0	1	1	1	1	0	0	0	1	0	0	1	0	0	0	0	0	0	0	1	0	1	1	0	1	0
34	0	1	1	1	1	0	1	1	1	1	0	0	0	1	0	0	1	1	0	1	0	0	0	0	1	0	1	1	0	1	0
35	0	1	1	1	1	0	1	1	1	1	0	0	0	1	0	0	0	0	0	0	0	0	0	0	1	0	1	1	0	1	0

CONSORT (Evaluated by researcher B)

No. \ item	1a	1b	2a	2b	3a	3b	4a	4b	5	5a	5b	5c	5d	6a	6b	7a	7b	8a	8b	9	10	11a	11b	11c	12a	12b	13a	13b	13c	14a	14b
38	0	1	1	1	1	0	1	1	1	1	0	0	0	1	0	0	0	0	0	0	0	0	0	0	1	0	1	0	0	0.5	0
39	0	1	1	1	1	0	1	1	1	1	0	0	0	1	0	0	0	0	0	0	0	0	0	0	1	0	1	0.5	0	1	0
40	0	1	1	1	1	0	1	1	1	1	0	0	0	0.5	0	0	0	0	0	0	0	0.5	0	0	1	0	1	0	0	1	0
41	0	1	1	1	1	0	1	1	1	1	1	1	0	1	0	0	1	1	0	1	0	1	0	0	1	1	1	1	0	1	0
42	0	1	1	1	1	0	1	1	1	1	0	0	0	1	0	0	0	1	0	0	0	0	0	0	1	0	1	0.5	0	0.5	0
43	0	1	1	1	1	0	1	1	1	1	1	1	0	1	0	0	0	0	0	0	0	0	0	0	1	0	1	1	0	1	0
44	0	1	1	1	1	0	1	1	1	1	0	0	0	1	0	0	0	0	0	0	0	0	0	0	1	0	1	1	0	1	0
45	0	1	1	1	1	0	1	1	1	1	0	0	0	1	0	0	0	0	0	0	0	0	0	0	1	0	1	1	0	1	0
46	0	1	0	1	1	0	1	1	1	1	0	0	0	0.5	0	0	0	0	0	0	0	0	0	0	0.5	0	1	0	0	0.5	0
47	0	1	1	1	1	0	1	1	1	1	0	0	0	0.5	0	0	1	1	0	1	0	0	0	0	0.5	0	1	1	0	1	0
48	0	1	1	1	1	0	1	1	1	1	1	0	0	1	0	0	0	1	0	0	0.5	0	0	1	1	0	1	0.5	0	0.5	0
49	0	1	1	1	1	0	1	1	1	1	0	0	0	1	0	0	0	0	0	1	0	0	0	0	1	0	1	0	0	0	0
50	0	1	1	1	1	0	1	1	1	1	0	0	0	1	0	0	0	0	0	0	0	0	0	0	1	0	1	0	0	1	0
51	0	1	1	1	1	0	1	1	1	1	0	0	0	1	0	0	0	1	0	0	0	0	0	0	1	0	1	1	0	1	0
52	0	1	1	1	1	0	1	1	1	1	0	0	0	1	0	0	0	0	0	0	0	0	0	0	1	0	1	0	0	1	0
53	0	1	1	1	1	0	1	1	1	1	0	0	0	0.5	0	0	0	1	0	0	0	0	0	0	0.5	0	1	0	0	0	0
54	0	1	1	1	1	0	1	1	1	1	0	0	0	1	0	0	1	1	0	0	0	0	0	0	1	0	1	0	0	0.5	0
55	0	1	1	1	1	0	1	1	1	1	0	0	0	1	0	0	0	0	0	0	0	0	0	0	1	0	1	0	0	0.5	0
56	0	1	1	1	1	0	1	1	1	1	0	0	0	1	0	0	0	1	0	0	0	0	0	0	1	0	1	0	0	1	0
57	0	1	1	1	1	0	1	1	1	1	0	0	0	1	0	0	1	1	0	0	0	0	0	0	1	0	1	0	0	0.5	0
58	0	1	1	1	1	0	1	1	1	1	0	0	0	1	0	0	0	0	0	0	0	0	0	0	1	0	1	0	0	0	0
59	1	1	1	1	1	0	1	1	1	1	1	0	0	1	0	0	1	1	0	1	0	1	0	0	1	0	1	1	0	1	0
60	0	1	1	1	1	0	1	1	1	1	0	0	0	1	0	0	0	1	0	0	0	0	0	0	1	0	1	0	0	0.5	0
61	0	1	1	1	1	0	1	1	1	1	0	0	0	1	0	0	0	0	0	0	0	0	0	0	1	0	1	0	0	0	0
62	0	1	1	1	1	0	1	1	1	1	0	0	0	1	0	0	0	1	1	1	0	0	0	0	1	0	1	0	0	1	0
63	0	1	1	1	1	0	1	1	1	1	0	0	0	0.5	0	0	0	1	0	1	0	0	0	0	0.5	0	1	0	0	1	0
64	0	1	0	1	0	0	1	1	1	1	0	0	0	0.5	0	0	0	0	0	0	0	0	0	0	0	0	1	0	0	0.5	0
65	1	1	1	1	0	0	1	1	1	1	0	0	0	1	0	0	0	1	0	1	0	0	0	0	1	0	1	0	0	1	0
66	1	1	1	1	1	0	1	1	1	1	0	0	0	0.5	0	1	1	1	0	1	0	0	0	0	0.5	0	1	0	0	1	0
67	0	1	1	1	1	0	1	1	1	1	0	0	0	0.5	0	0	0	0	0	0	0	0	0	0	0	0	1	0	0	0.5	0
68	0	1	1	1	1	0	1	1	1	1	1	0	0	1	0	0	0	1	0	0	0	0	0	0	1	0	1	0	0	0.5	0
69	0	1	1	1	1	0	1	1	1	1	0	0	0	1	0	0	0	1	0	0	0	0	0	0	1	0	1	0	0	0.5	0
70	0	1	1	1	1	0	1	1	1	1	0	0	0	1	0	0	0	1	0	0	0	0	0	0	1	0	1	0	0	1	0
71	0	1	1	1	1	0	1	1	1	1	0	0	0	1	0	0	0	1	0	0	0	0	0	0	1	0	1	0	0	1	0
72	0	1	1	1	1	0	1	1	1	1	0	0	0	1	0	0	0	0	0	0	0	0	0	0	1	0	1	0	0	1	0

CONSORT (Evaluated by researcher B)

item<br>No.	1a	1b	2a	2b	3a	3b	4a	4b	5	5a	5b	5c	5d	6a	6b	7a	7b	8a	8b	9	10	11a	11b	11c	12a	12b	13a	13b	13c	14a	14b
73	0	1	1	1	1	0	1	1	1	1	0	0	0	0.5	0	0	0	1	0	0	0	0	0	0	0.5	0	1	0	0	0.5	0
76	0	1	1	1	1	0	1	1	1	1	0	0	0	0.5	0	0	1	1	0	0	0	0	0	0	0.5	0	1	0	0	0.5	0
77	0	1	1	1	1	0	1	1	1	1	1	0	0	1	0	0	0	1	0	1	0	0.5	1	0	1	0	1	1	0	0.5	0
78	0	1	1	1	1	0	1	1	1	1	0	0	0	1	0	1	0	1	0	1	0.5	1	0	0	1	0	1	1	0	0.5	0
79	0	1	1	1	1	0	1	1	1	1	0	0	0	1	0	0	0	0	0	1	0	0.5	0	0	1	0	1	1	0	0.5	0
80	0	1	1	1	1	0	1	1	1	1	0	0	0	1	0	0	0	1	0	0	0		0	0	1	0	1	0	0	1	0
81	0	1	1	1	1	0	1	1	1	1	0	0	0	0.5	0	0	0	0	0	0	0	0	0	0	1	0	1	0	0	0.5	0
82	0	1	1	1	1	0	1	1	1	1	0	0	0	1	0	0	0	1	0	0	0	0	0	0	1	0	1	0	0	0.5	0
83	0	1	1	1	1	0	1	1	1	1	0	0	0	1	0	0	0	0	0	0	0	0	1	0	1	0	1	0	0	1	0
84	1	1	1	1	1	0	1	1	1	1	1	1	0	1	0	0	0	0	0	1	1	1	1	0	1	0	1	0	0	1	0
85	0	1	1	1	1	0	1	1	1	1	0	0	0	1	0	0	0	1	0	0	0	0	0	0	1	0	1	1	0	0.5	0
86	1	1	1	1	1	0	1	1	1	1	1	1	0	1	0	1	1	1	0	1	1	1	1	1	1	0	1	1	0	1	0
87	0	1	1	1	1	0	1	1	1	1	0	0	0	0.5	0	0	1	0	0	0	0	1	0	0	0.5	0	1	0	0	0.5	0
88	1	1	1	1	1	0	1	1	1	1	1	0	0	1	0	1	0	1	0	1	0	1	1	1	1	0	1	1	0	0.5	0
89	0	1	1	1	1	0	1	1	1	1	0	0	0	1	0	0	0	1	0	0	0	0	0	0	1	0	1	0	0	1	0
90	0	1	1	1	1	0	1	1	1	1	0	0	0	0.5	0	0	0	0	0	0	0	0	0	0	1	0	1	1	0	1	0
91	1	1	1	1	1	0	1	0	1	1	0	0	0	1	0	1	0	1	0	1	1	1	0	0	1	0	1	0.5	0	1	0
92	0	1	1	1	1	0	1	1	1	1	0	0	0	0.5	0	0	0	0	0	0	0	0	0	0	1	0	1	0	0	1	0
93	1	1	1	1	1	0	1	1	1	1	1	0	0	1	0	0	1	1	0	0	0	0	0	0	1	0	1	0	0	0.5	0
94	1	1	1	1	1	0	1	1	1	1	0	0	0	1	0	0	0	1	0	1	0	0	0	0	1	0	1	1	0	0.5	0
95	1	1	1	1	1	0	1	1	1	1	0	0	0	1	0	1	0	1	0	1	0	1	1	0	1	0	1	1	0	1	0
96	1	1	1	1	1	0	1	1	1	1	0	0	0	1	1	0	0	1	0	0	0	0	0	0	1	0	1	0	0	1	0
97	1	1	1	1	1	0	1	1	1	1	0	0	0	1	0	0	0	1	0	1	0	1	1	0	1	0	1	0.5	0	1	0
98	1	1	1	1	1	0	1	1	1	1	0	0	0	1	0	0	0	1	0	1	0	0	0	0	1	0	1	1	0	1	0
99	0	1	1	1	1	0	1	1	1	1	0	0	0	1	0	0	0	1	0	1	1	1	0	0	1	0	1	1	0	0.5	0
100	0	1	1	1	1	0	1	1	1	1	0	0	0	0.5	0	0	0	0	0	0	0	0	0	0	0.5	0	1	0	0	0	0
101	1	1	1	1	1	0	1	1	1	1	0	0	0	1	0	1	1	1	1	1	1	0	0	0	1	0	1	0.5	0	1	0
102	0	1	1	1	1	0	1	1	1	1	1	1	0	1	0	1	0	1	0	1	0	1	1	0	1	0	1	0.5	0	1	0

CONSORT (Evaluated by researcher B)

No. \ item	15	16	17a	17b	18	19	20	21	22	23	24	25
1	0.5	1	0	0	0	0	0	1	0.5	0	1	0
2	1	1	1	0	0	0	0	1	0.5	0	1	1
3	1	1	0	0	0	1	1	1	0.5	0	0	1
4	0.5	1	0	0	1	0	0	0	1	1	0	1
5	0.5	1	0	0	0	0	1	1	1	0	0	0
6	0.5	1	0	0	0	0	0	1	1	0	0	0
7	0.5	1	0	0	0	1	1	1	1	0	1	1
8	1	1	0	0	0	0	1	0	1	0	0	0
9	1	1	0	0	0	0	0	0	0.5	1	0	0
10	1	1	0	0	0	1	1	1	1	0	0	0
11	0.5	1	0	0	0	0	0	1	0.5	0	1	1
12	1	1	0	0	0	0	0	0	0	0	1	1
13	0.5	1	0	0	0	0	0	1	1	0	0	0
14	0.5	1	0	0	0	0	0	1	0.5	0	0	0
15	0.5	1	0	0	0	1	0	0	0.5	0	0	0
16	0.5	1	0	0	0	1	1	0	1	0	1	1
17	1	1	0	0	0	1	0	0	0.5	0	1	1
18	0	1	0	0	0	0	0	0	0.5	0	0	1
19	1	1	0	0	0	1	1	1	1	0	0	0
20	1	1	0	0	0	0	0	0	0.5	0	1	0
21	1	1	0	0	0	0	1	1	1	0	0	1
22	1	1	0	0	0	1	1	1	1	0	0	0
23	1	1	0	0	0	1	0	1	1	0	0	1
24	1	1	0	0	0	0	1	0	1	0	0	0
25	1	1	0	0	0	1	1	1	1	0	0	0
26	1	1	0	0	0	0	0	0	1	1	0	1
27	1	1	0	0	1	0	1	1	1	0	0	0
28	0.5	1	0	0	0	0	0	0	0.5	0	1	0
29	1	1	0	0	0	1	1	1	1	0	0	0
30	1	1	0	0	0	0	0	1	1	0	1	1
31	0.5	1	0	0	0	0	0	1	0.5	0	0	1
32	0.5	1	0	0	0	0	0	0	0.5	0	0	0
33	1	1	0	0	0	1	1	1	1	0	0	0
34	1	1	0	0	0	1	0	0	1	0	0	0
35	1	1	0	0	0	1	1	1	1	0	0	1

CONSORT (Evaluated by researcher B)

No.	item	15	16	17a	17b	18	19	20	21	22	23	24	25
38		1	1	0	0	0	1	0	1	0.5	0	1	0
39		0.5	1	0	0	0	0	0	0	0.5	0	0	0
40		0.5	1	0	0	0	0	1	0	1	0	1	0
41		1	1	0	0	0	1	1	1	1	0	0	0
42		1	1	0	0	0	0	0	0	1	0	0	1
43		1	1	0	0	0	0	1	1	1	0	1	1
44		1	1	0	0	0	1	1	1	1	0	0	1
45		1	1	0	0	0	1	1	0	1	0	0	1
46		0.5	1	0	0	0	0	0	1	0.5	0	0	0
47		0.5	1	0	0	0	0	0	1	0.5	0	0	1
48		1	1	0	0	0	0	0	1	0.5	0	0	0
49		1	1	0	0	0	1	1	0	1	0	0	1
50		1	1	0	0	0	0	0	1	1	0	0	0
51		1	1	0	0	0	1	1	0	1	0	0	1
52		1	1	0	0	0	0	0	1	0.5	0	0	0
53		0.5	1	0	0	0	1	0	0	0	0	0	1
54		0.5	1	0	0	0	0	0	1	0.5	0	0	0
55		0.5	1	0	0	0	0	0	1	0.5	0	0	0
56		1	1	0	0	0	0	1	0	1	0	0	0
57		1	1	0	0	0	0	1	1	1	0	0	0
58		0.5	1	0	0	0	0	0	1	1	0	0	0
59		1	1	0	0	0	1	0	0	0.5	0	0	0
60		0.5	1	0	0	0	0	0	1	0.5	0	0	1
61		0.5	1	0	0	0	1	0	0	0.5	0	0	0
62		0.5	1	0	0	0	0	1	1	1	0	0	0
63		0.5	1	0	0	0	0	0	1	0.5	0	0	1
64		0.5	1	0	0	0	1	0	1	0.5	0	0	1
65		0.5	1	0	0	0	0	0	0	0.5	0	1	0
66		1	1	0	0	0	0	1	0	1	0	0	0
67		0	1	0	0	0	0	0	0	0.5	0	0	0
68		0.5	1	0	0	0	0	0	1	0.5	0	0	0
69		0.5	1	0	0	0	1	0	1	1	0	1	1
70		1	1	0	0	0	0	0	1	0.5	0	0	0
71		0.5	1	0	0	0	1	0	1	0.5	0	1	0
72		0.5	1	0	0	0	0	0	1	0.5	0	0	0

CONSORT (Evaluated by researcher B)

No. \ item	15	16	17a	17b	18	19	20	21	22	23	24	25
73	1	1	0	0	0	0	0	1	1	0	1	1
76	1	1	0	0	0	0	1	1	1	0	0	0
77	1	1	0	0	0	1	1	0	1	1	1	1
78	1	1	0	0	0	0	0	0	0.5	1	1	1
79	1	1	0	0	0	0	0	0	1	1	1	1
80	0.5	1	0	0	0	1	0	0	0.5	0	0	0
81	1	1	0	0	0	1	1	0	1	0	1	1
82	0.5	1	0	0	0	0	0	0	0.5	0	1	1
83	1	1	0	0	0	1	0	1	0.5	0	1	0
84	1	1	0	0	0	1	1	1	1	1	1	1
85	0.5	1	0	0	0	0	0	1	0.5	0	1	1
86	1	1	0	0	0	1	1	1	1	1	1	1
87	1	1	0	0	0	1	1	1	1	0	0	0
88	1	1	1	0	0	1	1	0	1	1	1	1
89	0.5	1	0	0	0	0	0	1	0.5	0	1	1
90	1	1	0	0	0	1	0	1	1	0	1	0
91	1	1	0	0	0	1	1	0	1	1	0	0
92	0.5	1	0	0	0	0	0	1	0.5	0	0	1
93	1	1	0	0	0	0	0	0	0.5	0	1	1
94	1	1	0	0	0	0	0	0	0.5	1	1	1
95	1	1	1	0	0	1	1	0	1	1	1	1
96	1	1	0	0	0	1	0	1	0.5	0	1	1
97	1	1	1	0	0	1	1	0	1	0	1	0
98	1	1	0	0	0	1	1	1	1	0	0	0
99	1	1	0	0	0	1	1	0	1	0	0	0
100	1	1	0	0	0	0	1	1	1	0	0	0
101	0	1	0	0	0	1	0	1	1	0	1	1
102	1	1	0	0	0	0	1	0	1	0	0	1

STRICTA (Evaluated by researcher B)

No. \ item	1a	1b	1c	2a	2b	2c	2d	2e	2f	2g	3a	3b	4a	4b	5	6a	6b
1	1	1	0	1	1	0	0	0	1	0	1	1	1	0	1	0	1
2	1	1	0	1	0.5	1	1	1	1	0	1	1	0	0	0	0	1
3	1	1	0	1	1	1	1	1	1	1	1	1	0	0	0	0	1
4	1	1	0	1	0.5	1	0	1	1	1	1	1	0	0	1	1	1
5	1	1	0	1	1	1	1	1	1	1	1	1	1	1	0	0	1
6	1	1	0	1	1	1	1	1	1	1	1	1	1	0	0	0	1
7	1	1	0	1	1	1	0	1	1	1	1	1	1	0	1	0	1
8	1	1	0	1	1	1	1	1	1	1	1	1	0	0	0	1	1
9	1	1	0	1	0.5	1	1	1	1	1	1	1	1	0	1	0	1
10	1	1	0	1	1	0	1	1	1	1	1	1	1	0	0	0	1
11	1	1	1	1	0.5	0	0	0	0	0	1	1	0	1	0	1	1
12	1	1	0	1	0.5	1	0	1	1	1	1	1	0	0	0	0	1
13	1	0	1	1	1	0	1	1	1	1	1	1	1	1	0	0	1
14	1	1	0	1	0.5	1	1	1	1	0.5	1	1	0	0	0	0	1
15	1	1	1	1	0.5	0	0	1	1	0	1	1	1	1	0	0	1
16	1	1	0	1	0.5	0	1	1	1	1	1	1	0	0	0	1	1
17	1	0	0	1	1	0	1	1	1	1	1	1	0	0	0	1	1
18	1	1	0	1	1	0.5	0	1	1	0.5	1	1	0	0	0	0	1
19	1	1	0	1	0.5	1	1	1	1	1	1	1	0	1	0	1	1
20	1	1	0	1	0.5	1	1	1	1	0.5	1	1	0	0	0	0	1
21	1	1	0	1	1	1	1	1	1	1	1	1	0	0	0	1	1
22	1	1	0	1	1	1	1	1	1	1	1	1	0	0	0	1	1
23	1	1	0	1	1	1	1	1	1	1	1	1	0	0	0	1	1
24	1	1	0	1	1	1	1	1	1	1	1	1	0	0	0	1	1
25	1	1	0	1	1	0	1	1	1	1	1	1	0	0	0	0	1
26	1	1	0	1	0.5	1	1	1	1	1	1	1	0	1	1	1	1
27	1	1	0	1	1	1	0	1	1	1	1	1	0	0	1	1	1
28	1	1	0	1	1	1	1	1	1	1	1	1	0	0	0	0	0
29	1	1	0	1	1	1	1	1	1	1	1	1	1	0	1	1	1
30	1	1	0	1	1	1	1	1	1	1	1	1	0	0	0	0	1
31	1	1	1	1	1	1	1	1	1	1	1	1	1	0	0	0	1
32	1	1	1	1	1	1	0	1	1	0.5	1	1	1	0	0	0	1
33	1	1	0	1	0.5	0	0	1	1	1	1	1	0	0	0	0	1
34	1	1	1	1	1	1	1	1	1	1	1	1	1	0	0	0	1
35	1	1	0	1	1	1	1	1	1	1	1	1	0	0	0	1	1

STRICTA (Evaluated by researcher B)

No. \ item	1a	1b	1c	2a	2b	2c	2d	2e	2f	2g	3a	3b	4a	4b	5	6a	6b
36	1	1	0	1	1	1	0	1	1	1	1	1	0	0	0	1	1
37	1	1	0	1	1	1	1	1	1	1	1	1	0	0	0	1	1
38	1	0	1	1	0	0	0	1	1	0.5	1	1	0	0	0	0	1
39	1	1	1	1	1	1	0	1	1	1	1	1	1	0	0	0	1
40	1	1	0	1	1	0	1	1	1	1	1	1	0	0	0	1	1
41	1	1	0	1	1	1	1	1	1	0.5	1	1	0	1	1	0	1
42	1	0	1	1	1	1	1	1	1	1	1	1	0	0	0	0	1
43	1	1	0	1	1	1	0	1	1	1	1	1	0	0	0	0	1
44	1	1	0	1	1	1	1	1	1	1	1	1	0	0	0	1	1
45	1	1	0	1	1	1	0	1	1	1	1	1	0	0	0	0	1
46	1	0	1	1	0.5	0	0	1	1	0	1	1	0	0	0	0	1
47	1	1	1	1	0.5	0	0	0	1	0.5	1	1	0	0	0	0	1
48	1	1	1	1	1	1	1	1	1	1	1	1	0	0	0	1	1
49	1	1	0	1	1	1	1	1	1	1	1	1	0	0	0	0	1
50	1	1	0	1	1	1	1	1	1	1	1	1	0	0	0	0	1
51	1	1	0	1	1	1	0	1	1	1	1	1	0	0	0	0	1
52	1	0	1	1	1	1	1	1	1	0	1	1	0	0	0	0	1
53	1	0	0	1	1	0	0	1	0	0.5	1	1	0	1	0	1	1
54	1	1	1	1	0.5	0	0	1	1	0	1	1	0	0	0	1	1
55	1	0	0	1	0.5	0	1	0	1	1	1	1	0	0	0	0	1
56	1	1	0	1	1	1	1	1	1	0.5	1	1	0	0	0	1	1
57	1	1	0	1	1	0	1	1	1	1	1	1	0	0	0	0	1
58	1	0	0	1	0.5	0	1	1	1	0	1	1	0	0	0	0	1
59	1	1	1	1	1	1	1	1	1	1	1	1	0	0	0	0	1
60	1	0	0	1	0.5	1	1	1	1	1	1	1	0	0	0	0	1
61	1	1	1	1	0.5	0	1	1	1	0.5	1	1	0	0	0	0	1
62	1	1	0	1	1	1	1	1	1	0.5	1	1	0	0	0	0	1
63	1	1	0	1	0.5	0	0	1	1	1	1	1	0	0	0	0	1
64	1	0	1	1	1	1	0	1	1	0.5	1	1	1	0	0	0	1
65	1	1	0	1	0.5	1	0	1	1	1	1	1	0	0	0	0	1
66	1	1	0	1	1	0	0	1	1	1	1	1	0	0	0	0	1
67	1	0	0	1	1	0	0	0	1	0	1	1	1	0	0	0	1
68	1	1	0	1	1	1	1	1	1	0.5	1	1	0	0	0	1	1
69	1	0	1	1	1	0	0	0	1	1	1	1	1	0	1	1	1
70	1	0	0	1	1	1	0	1	1	1	1	1	1	0	1	0	1

STRICTA (Evaluated by researcher B)

item<br>No.	1a	1b	1c	2a	2b	2c	2d	2e	2f	2g	3a	3b	4a	4b	5	6a	6b
71	1	1	0	1	1	1	0	1	1	1	1	1	1	0	0	0	1
72	1	1	0	1	1	1	0	1	1	0.5	1	1	0	1	0	0	1
73	1	1	0	1	1	1	0	1	1	1	1	1	1	0	0	0	1
74	1	1	1	1	0.5	1	0	1	0	0	1	1	1	0	0	0	1
75	1	1	0	1	1	1	1	1	1	1	1	1	0	0	0	1	1
76	1	1	0	1	1	0	1	1	1	1	1	1	0	0	0	0	1
77	1	0	0	1	0.5	1	1	1	1	1	1	1	1	0	1	1	1
78	1	1	0	1	0.5	1	0	1	1	0.5	1	1	1	0	1	0	1
79	1	1	0	1	0.5	1	1	0	1	0.5	1	1	1	0	1	0	1
80	1	1	0	1	0.5	0	1	1	1	0.5	1	1	0	0	0	0	1
81	1	1	0	1	1	1	1	1	1	0.5	1	1	0	0	0	1	1
82	1	1	0	1	0.5	1	1	1	1	1	1	1	0	0	0	0	1
83	1	0	1	1	1	1	1	1	1	1	1	1	0	0	0	0	1
84	1	1	0	1	1	1	1	1	1	1	1	1	0	1	1	1	1
85	1	1	1	1	1	0	1	1	1	1	1	1	0	0	0	0	1
86	1	1	0	1	1	1	1	1	1	0	1	1	0	0	1	1	1
87	1	1	0	1	1	1	0	1	1	1	1	1	0	0	0	1	1
88	1	1	0	1	0.5	1	1	1	1	1	1	1	1	1	1	1	1
89	1	0	1	1	1	1	0	0	1	0	1	1	1	0	0	0	1
90	1	1	0	1	1	1	1	1	1	0	1	1	0	1	1	0	1
91	1	1	0	1	1	0	1	0	1	1	1	1	0	1	0	1	1
92	1	0	1	1	0.5	1	1	1	1	1	1	1	1	0	0	0	1
93	1	0	1	1	0.5	1	0	1	1	0.5	1	1	0	0	0	1	1
94	1	0	0	1	0.5	1	1	1	1	0.5	1	1	0	0	1	1	1
95	1	1	0	1	1	1	1	1	1	1	1	1	0	1	1	1	1
96	1	1	1	1	0.5	1	1	1	1	1	1	1	0	0	0	0	1
97	1	1	0	1	1	1	0	0	1	1	1	1	1	0	0	1	1
98	1	1	0	1	0	1	1	1	1	1	1	1	0	0	0	1	1
99	1	1	1	0	1	0	1	1	1	1	1	1	0	0	0	1	1
100	1	1	0	1	1	1	1	1	1	1	1	1	0	0	0	1	1
101	1	1	1	1	0.5	1	1	1	1	1	1	1	0	1	1	0	1
102	1	1	0	1	1	0	0	0	1	1	1	1	0	1	1	0	1
