# Supplementary material for: Assessment of Reporting Quality in Randomized Controlled Trials of Acupuncture for Primary Insomnia with CONSORT Statement and STRICTA Guidelines
Source: Evid Based Complement Alternat Med. 2022 Feb 17;2022:5157870. doi: 10.1155/2022/5157870 (PMC8872660; doi:10.1155/2022/5157870)
Supplement: Supplementary Materials — S1: search strategies in this paper. S2: evaluation records (CONSORT and STRICTA) by researchers. S3: the information of included papers. S4: the CONSORT checklist. S5: the STRICTA checklist. S6: the total scores for CONSORT and STRICTA. [file 5157870.f1.zip › 5157870.f1/S6 Total scores for CONSORT and STRICTA.pdf]

No.	total score for CONSORT	total score for STRICTA	year
1	15.5	10	2021
2	24	10.5	2020
3	22.5	12	2020
4	23	12.5	2020
5	17	12.5	2020
6	16	12.5	2020
7	22.5	14	2020
8	17.5	12	2020
9	21.5	13.5	2020
10	23	12	2020
11	16	8.5	2020
12	17	10.5	2020
13	14.5	13	2020
14	15	11	2020
15	14.5	10.5	2020
16	19	11.5	2019
17	20	12	2019
18	15.5	10	2019
19	29	13.5	2019
20	16	11	2019
21	20	13	2019
22	18	13	2019
23	20	13	2019
24	23	13	2019
25	20.5	11	2019
26	25	14.5	2019
27	26	14	2019
28	15.5	11	2019
29	25.5	13.5	2019
30	18.5	12	2018
31	18.5	14	2018
32	14	12.5	2018
33	20	9.5	2018
34	21	14	2018
35	20	13	2018
36	19	11.5	2018
37	19	13	2018
38	17	9.5	2017
39	13	13	2017
40	17.5	12	2017
41	27	12.5	2017
42	17	11	2017
43	20	11	2018
44	21.5	13	2017
45	21	11	2017
46	12.5	8.5	2016
47	20	10	2016
48	19	13.5	2016
49	20	12	2016
50	16	12	2016
51	20	11	2016
52	15	11	2015
53	15	8.5	2015
54	16.5	10.5	2015
55	14.5	8.5	2014
56	18	12.5	2014
57	18.5	11	2014

No.	total score for CONSORT	total score for STRICTA	year
58	14	8.5	2013
59	24.5	12.5	2013
60	16.5	10.5	2011
61	13	11	2011
62	19.5	11.5	2009
63	18	9.5	2008
64	14	11.5	2008
65	16.5	10.5	2007
66	19.5	10	2007
67	11.5	10	2006
68	15.5	10.5	2020
69	18.5	10	2020
70	16.5	11	2020
71	19	12	2019
72	15	10.5	2018
73	17.5	12	2013
74	17	10.5	2020
75	14.5	13	2006
76	18	11	2015
77	23.5	13.5	2021
78	22	13	2021
79	21.5	12	2021
80	17	11	2009
81	18	12.5	2008
82	16.5	11.5	2015
83	17.5	12	2009
84	27	15	2020
85	19.5	11	2019
86	32	14	2017
87	18	12	2016
88	29	15.5	2013
89	18	10	2014
90	19.5	13	2012
91	24.5	13	2009
92	15.5	12.5	2011
93	19	11	2019
94	21	12	2020
95	28	15	2020
96	21.5	11.5	2015
97	23.5	11.5	2008
98	22	12	2010
99	21.5	11.5	2017
100	16	13	2009
101	24	14.5	2019
102	24.5	11	2008
