# Supplementary material for: Assessment of Reporting Quality in Randomized Controlled Trials of Acupuncture for Primary Insomnia with CONSORT Statement and STRICTA Guidelines
Source: Evid Based Complement Alternat Med. 2022 Feb 17;2022:5157870. doi: 10.1155/2022/5157870 (PMC8872660; doi:10.1155/2022/5157870)
Supplement: Supplementary Materials — S1: search strategies in this paper. S2: evaluation records (CONSORT and STRICTA) by researchers. S3: the information of included papers. S4: the CONSORT checklist. S5: the STRICTA checklist. S6: the total scores for CONSORT and STRICTA. [file 5157870.f1.zip › 5157870.f1/S5 STRICTA-2010-Checklist.doc]

***Citation:*** *MacPherson H, Altman DG, Hammerschlag R, Youping L, Taixiang W, White A, Moher D; STRICTA Revision Group. Revised STandards for Reporting Interventions in Clinical Trials of Acupuncture (STRICTA): extending the CONSORT statement. PLoS Med. 2010 Jun 8;7(6):e1000261*

**Table 1: STRICTA 2010 checklist of information to include when reporting interventions in a clinical trial of acupuncture (Expansion of Item 5 from CONSORT 2010 checklist)**

| **Item** | **Detail** |
| --- | --- |
| **1. Acupuncture rationale** | 1a) Style of acupuncture (e.g. Traditional Chinese Medicine, Japanese, Korean, Western medical, Five Element, ear acupuncture, etc) |
|  | 1b) Reasoning for treatment provided, based on historical context, literature sources, and/or consensus methods, with references where appropriate |
|  | 1c) Extent to which treatment was varied |
| **2. Details of needling** | 2a) Number of needle insertions per subject per session (mean and range where relevant) |
|  | 2b) Names (or location if no standard name) of points used (uni/bilateral) |
|  | 2c) Depth of insertion, based on a specified unit of measurement, or on a particular tissue level |
|  | 2d) Response sought (e.g. *de qi* or muscle twitch response) |
|  | 2e) Needle stimulation (e.g. manual, electrical) |
|  | 2f) Needle retention time |
|  | 2g) Needle type (diameter, length, and manufacturer or material) |
| **3. Treatment regimen** | 3a) Number of treatment sessions |
|  | 3b) Frequency and duration of treatment sessions |
| **4. Other components of treatment** | 4a) Details of other interventions administered to the acupuncture group (e.g. moxibustion, cupping, herbs, exercises, lifestyle advice) |
|  | 4b) Setting and context of treatment, including instructions to practitioners, and information and explanations to patients |
| **5. Practitioner background** | 5) Description of participating acupuncturists (qualification or professional affiliation, years in acupuncture practice, other relevant experience) |
| **6. Control or comparator interventions** | 6a) Rationale for the control or comparator in the context of the research question, with sources that justify this choice |
|  | 6b) Precise description of the control or comparator. If sham acupuncture or any other type of acupuncture-like control is used, provide details as for Items 1 to 3 above. |

Note: This checklist, which should be read in conjunction with the explanations of the STRICTA items provided in the main text, is designed to replace CONSORT 2010’s item 5 when reporting an acupuncture trial.

**Table 2: CONSORT 2010 checklist with the Non-pharmacological Trials Extension to CONSORT (with STRICTA 2010 extending CONSORT Item 5 for acupuncture trials)**

| **Section/Topic** | **Item #** | **CONSORT 2010 Statement*: Checklist item[10]. Describe:** | **Additional items from the Non-pharmacological Trials Extension to CONSORT[14]. Add:** |
| --- | --- | --- | --- |
| *TITLE AND ABSTRACT* |  |  |  |
|  | 1.a | Identification as a randomized trial in the title | In the abstract, description of the experimental treatment, comparator, care providers, centres and blinding status. |
|  | 1.b | Structured summary of trial design, methods, results, and conclusions; for specific guidance see CONSORT for Abstracts [58,59] |  |
| *INTRODUCTION* |  |  |  |
| Background and objectives | 2.a | Scientific background and explanation of rationale |  |
|  | 2.b | Specific objectives or hypotheses |  |
| METHODS |  |  |  |
| Trial design | 3.a | Description of trial design (e.g., parallel, factorial) including allocation ratio |  |
|  | 3.b | Important changes to methods after trial commencement (e.g. eligibility criteria), with reasons |  |
| Participants | 4.a | Eligibility criteria for participants | When applicable, eligibility criteria for centers and those performing the interventions. |
|  | 4.b | Settings and locations where the data were collected |  |
| Interventions | **5** | **The interventions for each group with sufficient details to allow replication, including how and when they were actually administered** | **Precise details of both the experimental treatment and comparator - see Table 1 for details** |
| Outcomes | 6.a | Completely defined pre-specified primary and secondary outcome measures, including how and when they were assessed |  |
|  | 6.b | Any changes to trial outcomes after the trial commenced with reasons |  |
| Sample size | 7.a | How sample size was determined | When applicable, details of whether and how the clustering by care providers or centers was addressed. |
|  | 7.b | When applicable, explanation of any interim analyses and stopping guidelines |  |
| Randomization |  |  |  |
| *Sequence generation* | 8.a | Method used to generate the random allocation sequence | When applicable, how care providers were allocated to each trial group. |
|  | 8.b | Type of randomization; details of any restriction (e.g., blocking and block size) |  |
| *Allocation concealment* | 9 | Mechanism used to implement the random allocation sequence (e.g., sequentially numbered containers), describing any steps taken to conceal the sequence until interventions were assigned |  |
| *Implementation* | 10 | Who generated the random allocation sequence, who enrolled participants, and who assigned participants to interventions |  |
| Blinding | 11.a | If done, who was blinded after assignment to interventions (e.g. participants, care providers, those assessing outcomes) and how | Whether or not those administering co-interventions were blinded to group assignment. If blinded, method of blinding and description of the similarity of interventions. |
|  | 11.b | If relevant, description of the similarity of interventions |  |
| Statistical methods | 12.a | Statistical methods used to compare groups for primary and secondary outcomes | When applicable, details of whether and how the clustering by care providers or centers was addressed. |
|  | 12.b | Methods for additional analyses, such as subgroup analyses and adjusted analyses |  |
| RESULTS |  |  |  |
| Participant flow (A diagram is strongly recommended) | 13.a | For each group, the numbers of participants who were randomly assigned, received intended treatment, and were analyzed for the primary outcome | The number of care providers or centers performing the intervention in each group and the number of patients treated by each care provider or in each center. |
|  | 13.b | For each group, losses and exclusions after randomization, together with reasons |  |
| Implementation of intervention |  |  | Details of the experimental treatment and comparator as they were implemented. |
| Recruitment | 14.a | Dates defining the periods of recruitment and follow-up |  |
|  | 14.b | Why the trial ended or was stopped |  |
| Baseline data | 15 | A table showing baseline demographic and clinical characteristics for each group | When applicable, a description of care providers (case volume, qualification, expertise, etc.) and centers (volume) in each group. |
| Numbers analyzed | 16 | For each group, number of participants (denominator) included in each analysis and whether the analysis was by original assigned groups |  |
| Outcomes and estimation | 17.a | For each primary and secondary outcome, results for each group, and the estimated effect size and its precision (e.g., 95% confidence interval) |  |
|  | 17.b | For binary outcomes, presentation of both absolute and relative effect sizes is recommended |  |
| Ancillary analyses | 18 | Results of any other analyses performed, including subgroup analyses and adjusted analyses, distinguishing pre-specified from exploratory |  |
| Harms | 19 | All important harms or unintended effects in each group; for specific guidance see CONSORT for Harms [60] |  |
| DISCUSSION |  |  |  |
| Limitations | 20 | Trial limitations, addressing sources of potential bias, imprecision, and, if relevant, multiplicity of analyses |  |
| Generalizability | 21 | Generalizability (external validity, applicability) of the trial findings | Generalizability (external validity) of the trial findings according to the intervention, comparators, patients and care providers and centers involved in the trial. |
| Interpretation | 22 | Interpretation consistent with results, balancing benefits and harms, and considering other relevant evidence | In addition, take into account the choice of the comparator, lack of or partial blinding, unequal expertise of care providers or centers in each group. |
| *Other Information* |  |  |  |
| Registration | 23 | Registration number and name of trial registry |  |
| Protocol | 24 | Where the full trial protocol can be accessed, if available |  |
| Funding | 25 | Sources of funding and other support (e.g., supply of drugs); role of funders |  |

* We strongly recommend reading this Statement in conjunction with the CONSORT 2010 explanation and elaboration [11] for important clarifications on all the items. If relevant, we also recommend reading CONSORT extensions for cluster randomized trials [61], noninferiority and equivalence trials [62], herbal interventions [63], and pragmatic trials [16]. Moreover, additional extensions are forthcoming. For those and also for up-to-date references relevant to this checklist, see http://www.consort-statement.org.
